# Overexpression of CHKA contributes to tumor progression and metastasis and predicts poor prognosis in colorectal carcinoma

**DOI:** 10.18632/oncotarget.11433

**Published:** 2016-08-20

**Authors:** Liang Hu, Ruo-Yu Wang, Jian Cai, Dan Feng, Guang-Zhen Yang, Qing-Guo Xu, Yan-Xia Zhai, Yu Zhang, Wei-Ping Zhou, Qing-Ping Cai

**Affiliations:** ^1^ Anal-Colorectal Surgery Institute, 150th Hospital of PLA, Luoyang, China; ^2^ The Third Department of Hepatic Surgery, Eastern Hepatobiliary Surgery Hospital, Second Military Medical University, Shanghai, China; ^3^ Department of Colorectal Surgery, The Sixth Affiliated Hospital, Sun Yat-sen University, Guangzhou, China; ^4^ Department of Oncology, Changhai Hospital, Second Military Medical University, Shanghai, China; ^5^ Department of Clinical Laboratory, 150th Hospital of PLA, Luoyang, China; ^6^ Department of Gastrointestine Surgery, Changzheng Hospital, Second Military Medical University, Shanghai, China

**Keywords:** CHKA, colorectal carcinoma, progression, prognosis, biomarker

## Abstract

Aberrant expression of choline kinase alpha (CHKA) has been reported in a variety of human malignancies including colorectal carcinoma (CRC). However, the role of CHKA in the progression and prognosis of CRC remains unknown. In this study, we found that CHKA was frequently upregulated in CRC clinical samples and CRC-derived cell lines and was significantly correlated with lymph node metastasis (*p* = 0.028), TNM stage (*p* = 0.009), disease recurrence (*p* = 0.004) and death (*p* < 0.001). Survival analyses indicated that patients with higher CHKA expression had a significantly shorter disease-free survival (DFS) and disease-specific survival (DSS) than those with lower CHKA expression. Multivariate analyses confirmed that increased CHKA expression was an independent unfavorable prognostic factor for CRC patients. In addition, combination of CHKA with TNM stage was a more powerful predictor of poor prognosis than either parameter alone. Functional study demonstrated that knockdown of CHKA expression profoundly suppressed the growth and metastasis of CRC cells both *in vitro* and *in vivo*. Mechanistic investigation revealed that EGFR/PI3K/AKT pathway was essential for mediating CHKA function. In conclusion, our results provide the first evidence that CHKA contributes to tumor progression and metastasis and may serve as a novel prognostic biomarker and potential therapeutic target in CRC.

## INTRODUCTION

Colorectal carcinoma (CRC) is the third most commonly diagnosed cancer in males and the second in females worldwide. According to data from the International Agency for Research on Cancer, there were an estimated 1.4 million new cases and 693,900 deaths occurring in 2012 [1]. Although the survival of CRC patients has slowly but steadily improved during the past decades in the developed countries, increases in CRC mortality rates have been observed in several historically low-risk regions including China [2, 3]. The main cause of death in CRC patients is tumor progression and metastasis, however, the underlying molecular events contributing to CRC progression and metastasis are still not fully understood. In addition, although classification according to TNM stage provides valuable prognostic information and guides therapy decisions for CRC patients, clinical outcome varies greatly even among patients of the same TNM stage category. Therefore, it is urgent needed to search for novel prognostic biomarkers and therapeutic targets for patients with CRC.

Aberrant choline phospholipid metabolism is a metabolic hallmark of cancer that has been implicated in tumorigenesis and progression [4]. Among the multiple enzymes involved in the biosynthesis of phosphatidylcholine, the major structural component of eukaryotic cell membranes, choline kinase is the first enzyme of the Kennedy pathway responsible for catalyzing the phosphorylation of free choline to form phosphocholine [5, 6]. In mammalian cells, choline kinase is encoded by two separate genes, choline kinase alpha (CHKA) and beta (CHKB), of which only CHKA has a central role in sustaining the biosynthesis of phosphatidylcholine [7, 8]. To date, Increased expression and enzymatic activity of CHKA has been identified in a variety of human malignancies including breast, lung, colorectal, bladder, prostate, ovarian, endometrial carcinomas, osteosarcoma, and T-cell lymphoma [9–18]. In addition to its metabolic function, CHKA has been proven to play a critical role in tumorigenesis, cancer progression and metastasis of several cancers, establishing it as an oncogene [4, 14, 18–22]. Encouragingly, selective inhibition of CHKA either by siRNA-mediated gene silencing or small molecule pharmacological inhibitors exhibits effective antitumoral activity both *in vitro* and *in vivo* [12, 14, 18, 21–32]. These characteristics indicate that CHKA has pleiotropic effects in driving cancer development and progression which can be exploited as a potential novel oncotarget. Meanwhile, the prognostic significance of CHKA overexpression in human malignant diseases has also been revealed. Ramírez de Molina *et al.* reported that overexpression of CHKA independently predicts poor survival in patients with early-stage non-small cell lung cancer (NSCLC) [33]. Likewise, Kwee *et al.* also observed a significant association between intratumoral CHKA expression and increased mortality in hepatocellular carcinoma (HCC) patients [34].

Despite these findings, however, the clinicopathologic significance and biological relevance of CHKA in the progression of CRC has not been investigated to date. In the present study, we examined both the mRNA and protein expression levels of CHKA in CRC-derived cell lines and clinical samples and analyzed the correlation of CHKA expression with clinicopathologic features and with patient survival in a CRC cohort. In addition, we explored the potential role of CHKA in the proliferation and metastasis of CRC cells *in vitro* and *in vivo*. Our results provide the first evidence that increased CHKA expression contributes to aggressive behaviors of CRC cells and correlates with tumor progression and metastasis and may serve as an independent unfavorable prognostic indicator for CRC patients.

## RESULTS

### Increased CHKA expression correlates with aggressive clinicopathologic features of CRC patients

**Figure 1:**
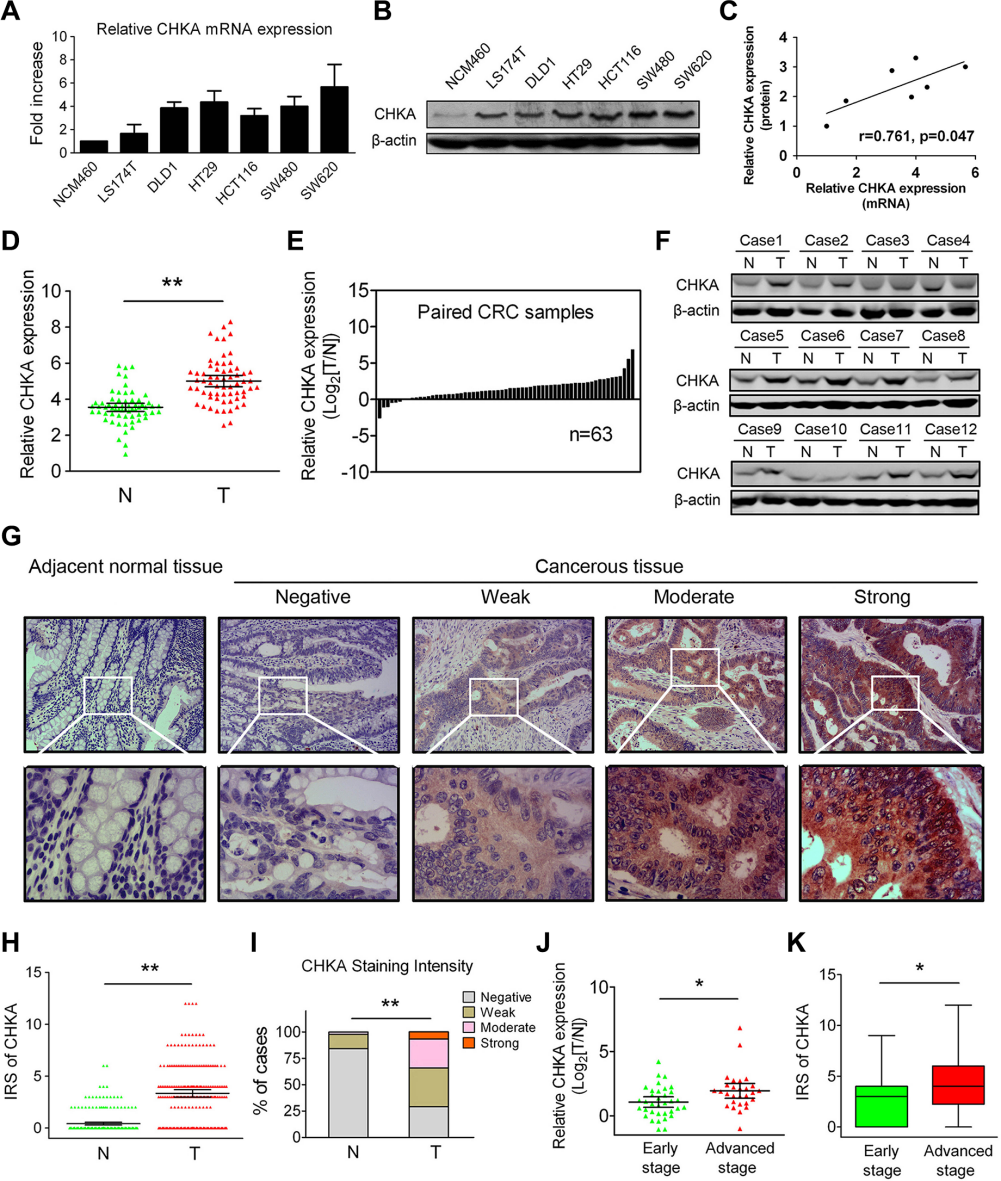
Increased expression levels of CHKA in CRC cell lines and clinical samples (**A**) Relative expression levels of CHKA mRNA in NCM460 and CRC-derived cell lines were determined by real-time qPCR methods. Gene expression results were normalized by internal control β-actin. (**B**) Protein expression levels of CHKA in NCM460 and CRC-derived cell lines were determined by western blot assay. β-actin was used as a loading control. (**C**) Correlations between relative expression levels of CHKA mRNA and protein in the cell lines examined. (**D–E**) Relative expression levels of CHKA mRNA in 63 paired human primary CRC tissues and adjacent nontumor tissues were determined by real-time qPCR. Gene expression results were normalized by β-actin. (T, tumor tissues; N, adjacent nontumor tissues) (**F**) CHKA protein expression in paired tumor and adjacent nontumor tissues was determined by western blot assay. (**G**) Representative immunohistochemical expression patterns of CHKA in cancerous and adjacent normal mucosa tissues are shown. (Magnification, upper panel, ×100; lower panel, ×400) (**H**) Comparison of IRS values of CHKA expression in 234 paired primary CRC tissues and adjacent nontumor tissues. (**I**) Percentage of cases with different staining intensity of CHKA in the tumor or adjacent nontumor tissues in the study cohort. (**J**) Comparison of the expression levels of CHKA mRNA in the 63 fresh-frozen CRC tissue samples from patients with different clinical stages (early stage, *n* = 35; advanced stage, *n* = 28). (**K**) Comparison of the IRS values of CHKA expression in the 234 CRC tissue samples from patients with different clinical stages (early stage, *n* = 146; advanced stage, *n* = 88). *p < 0.05; **p < 0.01.

We first examined the mRNA and protein expression levels of CHKA in several human CRC-derived cell lines (LS174T, DLD1, HT29, HCT116, SW480, and SW620) and the normal colon epithelial cell line NCM460. Real-time quantitative polymerase chain reaction (qPCR) and western blot analyses revealed that both the mRNA and protein expression levels of CHKA were markedly increased in all six CRC cell lines examined when compared to the NCM460 cells (Figure 1A and 1B). In addition, the relative expression of CHKA mRNA and protein in the cell lines investigated was positively correlated (Figure 1C). Then, we detected CHKA mRNA expression in 63 paired primary CRC tissues and corresponding adjacent nontumor samples. The qPCR results showed that the relative expression of CHKA mRNA was significantly higher in the cancerous tissues than adjacent non-cancerous tissues (Figure 1D, *p* < 0.01), with 87.3% (55/63) of the CRC tissue specimens tested showed a higher expression level of CHKA mRNA when compared to matched non-cancerous counterparts (Figure 1E). Similar results were also observed in the western blot analysis (Figure 1F).

To further determine the protein phenotypic expression patterns of CHKA in CRC clinical samples, immunohistochemical analysis was performed in 234 paired paraffin-embedded CRC specimens. Each pair consisted of cancerous and adjacent non-cancerous tissues derived from the same patient. The representative immunostainings of CHKA protein (negative, weak, moderate, strong) in CRC tissues were shown in Figure 1G, and positive staining was observed mainly in the cytoplasm. The immunohistochemical data clearly showed that the immunoreactive score (IRS) values of CHKA were significantly higher in tumor tissues (Figure 1H, *p* < 0.01). Positive staining of CHKA was detected in 70.9% (166/234) of the cancerous samples. Among them, 36.8% (86/234), 27.3% (64/234) and 6.8% (16/234) of the cases show weak, moderate, and strong staining of CHKA protein, respectively. In striking contrast, among the adjacent normal mucosa tissues examined, 84.2% (197/234) of the cases showed negative staining, 13.7% (32/234) of the cases showed weak staining, 2.1% (5/234) of cases showed moderate staining, while none of the cases showed strong staining of CHKA (Figure 1I, *p* < 0.01). Moreover, CHKA expression was significantly upregulated in advanced stage (stage III) CRC tissues compared with early stage (stages I–II) tumor counterparts at both the mRNA and protein levels (Figure 1J and 1K, all *p* < 0.05). These findings are in keeping with previous reports [10, 35] and definitely confirm that CHKA expression is frequently upregulated in CRC.

To evaluate the association of CHKA expression levels with clinicopathologic characteristics, the 234 patients in the study cohort were divided into high and low CHKA expression subgroups with its median IRS value as the cut-off. As shown in Table 1, high levels of CHKA protein in tumors were significantly correlated with increased lymph node metastasis (*p* = 0.028), advanced TNM stage (*p* = 0.009), increased disease recurrence (*p* = 0.004), and death (*p* < 0.001). While, no significant correlations were observed between the expression of CHKA and gender (*p* = 0.369), age (*p* = 0.228), tumor location (*p* = 0.155), tumor differentiation grade (*p* = 0.074), tumor size (*p* = 0.911), local invasion (*p* = 0.077), or adjuvant chemotherapy (*p* = 0.104). Spearman rank correlation analysis further revealed that intratumoral CHKA expression was positively correlated with local invasion (*r* = 0.148, *p* = 0.024), lymph node metastasis (*r* = 0.144, *p* = 0.028), TNM stage (*r* = 0.187, *p* = 0.004), disease recurrence (*r* = 0.186, *p* = 0.004), and death (*r* = 0.239, *p* < 0.001) (Table S1). Collectively, these findings indicate that upregulated CHKA expression may be linked with malignant progression of CRC.

**Table 1:** Association between CHKA protein expression and clinicopathologic characteristics of CRC patients in the study cohort

Characteristics	No. of patients (%) (*n* = 234)	CHKA expression		*P* value^a^
		Low (%) (*n* = 79)	High (%) (*n* = 155)	
**Gender**				0.369
Female	103 (44.0)	38 (48.1)	65 (41.9)	
Male	131 (56.0)	41 (51.9)	90 (58.1)	
**Age (years)**				0.228
< 60	68 (29.1)	19 (24.1)	49 (31.6)	
≥ 60	166 (70.9)	60 (75.9)	106 (68.4)	
**Tumor location**				0.155
Rectum	104 (44.4)	30 (38.0)	74 (47.7)	
Colon	130 (55.6)	49 (62.0)	81 (52.3)	
**Differentiation grade**				0.074
Well	21 (9.0)	11 (13.9)	10 (6.5)	
Moderate	157 (67.1)	54 (68.4)	103 (66.4)	
Poor	56 (23.9)	14 (17.7)	42 (27.1)	
**Tumor size (cm)**				0.911
< 5	93 (39.7)	31 (39.2)	62 (40.0)	
≥ 5	141 (60.3)	48 (60.8)	93 (60.0)	
**Local invasion**				0.077
pT_1_-T_2_	38 (16.2)	18 (22.8)	20 (12.9)	
pT_3_	168 (71.8)	55 (69.6)	113 (72.9)	
pT_4_	28 (12.0)	6 (7.6)	22 (14.2)	
**Lymph node metastasis**				**0.028**
Negative	146 (62.4)	57 (72.2)	89 (57.4)	
Positive	88 (37.6)	22 (27.8)	66 (42.6)	
**TNM stage**				**0.009**
I	33 (14.1)	18 (22.8)	15 (9.7)	
II	113 (48.3)	39 (49.4)	74 (47.7)	
III	88 (37.6)	22 (27.8)	66 (42.6)	
**Adjuvant chemotherapy**				0.104
No	110 (47.0)	43 (54.4)	67 (43.2)	
Yes	124 (53.0)	36 (45.6)	88 (56.8)	
**Recurrence**				**0.004**
No	103 (44.0)	45 (57.0)	58 (37.4)	
Yes	131 (56.0)	34 (43.0)	97 (62.6)	
**Death**				**< 0.001**
No	133 (56.8)	58 (73.4)	75 (48.4)	
Yes	101 (43.2)	21 (26.6)	80 (51.6)	

a Pearson chi-square test was used for comparison between subgroups. Bold type indicates statistical significance.

### CHKA upregulation predicts poor prognosis in patients with CRC

**Figure 2:**
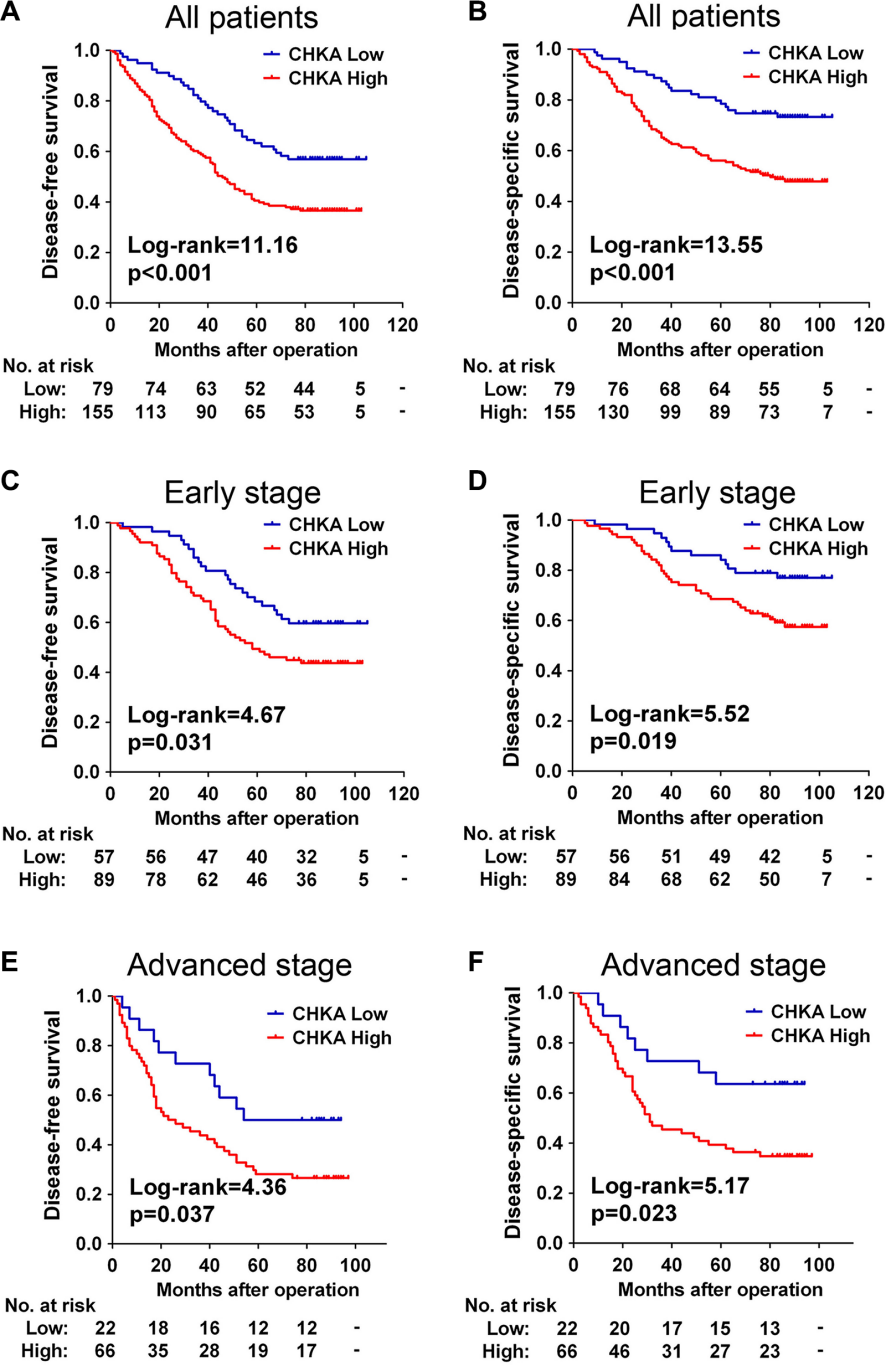
CHKA upregulation predicts poor prognosis in patients with CRC (**A–B**) Kaplan-Meier curves for disease-free survival (A) and disease-specific survival (B) of all CRC patients in the study cohort according to CHKA expression status. (**C–D**) Kaplan-Meier curves for disease-free survival (C) and disease-specific survival (D) of patients with early stage tumors according to CHKA expression status. (**E–F**) Kaplan-Meier curves for disease-free survival (E) and disease-specific survival (F) of patients with advanced stage tumors according to CHKA expression status. The *p*-value was determined using the log-rank test. The absolute number of patients at risk in each subgroup was listed below.

We next assessed the prognostic value of intratumoral CHKA expression in the 234 CRC patients. At the final follow-up in March 2015, 56.0% (131/234) of the patients suffered recurrence, and 43.2% (101/234) died. The distribution of DFS and DSS times was shown in Table S2 and S3. Kaplan-Meier analysis revealed that patients in the high CHKA group had a significantly shorter DFS (*p* < 0.001) and DSS (*p* < 0.001) than those in the low CHKA group (Figure 2A and 2B). The cumulative 5-year DFS and DSS rates were 63.3% and 78.5% in patients with low-CHKA tumors, whereas it was only 40.5% and 56.1% in those with high-CHKA tumors, respectively. In our cohort, patients who had advanced stage tumors had a significantly unfavorable prognosis compared with those who had early stage tumors (Figure S1, all *p* < 0.001).

To assess whether CHKA expression represents an independent prognostic indicator in CRC, the effect of each variable on survival was determined by the Cox regression analysis. In Cox univariate regression analysis, CHKA expression was significantly associated with DFS (*p* = 0.001) and DSS (*p* < 0.001). After adjustment for all the potential confounding factors that may have a clinical impact on progression or survival for this disease, Cox multivariate regression analysis revealed that high CHKA expression (*p* = 0.002), together with advanced TNM stage (*p* = 0.037), was independently associated with poor DFS, while CHKA expression (*p* = 0.001) and TNM stage (*p* = 0.006) were also independent risk factors for DSS (Table 2).

**Table 2:** Univariate and multivariate analyses of CHKA expression and patients' survival in the study cohort

Variables	Categories	Univariate analysis			Multivariate analysis		
		HR	95% CI	*P* value^a^	HR	95% CI	*P* value^a^
**Disease-free survival**							
Gender	Male/female	1.042	0.738–1.471	0.817	1.052	0.730–1.515	0.785
Age (years)	≥ 60/< 60	1.228	0.828–1.822	0.308	1.465	0.953–2.253	0.082
Tumor location	Colon / rectum	1.141	0.807–1.615	0.455	1.211	0.820–1.789	0.337
Tumor size (cm)	≥ 5/< 5	1.240	0.869–1.769	0.236	1.203	0.829–1.744	0.330
Differentiation grade	Poor / well + moderate	1.437	0.976–2.117	0.066	1.337	0.787–2.273	0.283
Local invasion	pT3-4/pT1-2	2.012	1.156–3.504	**0.013**	1.416	0.787–2.547	0.245
Lymph node metastasis ^b^	Positive /negative	1.902	1.346–2.687	**< 0.001**			
TNM stage	III/I + II	1.902	1.346–2.687	**< 0.001**	1.885	1.038–3.421	**0.037**
Adjuvant chemotherapy	Yes/no	1.761	1.242–2.498	**0.001**	0.943	0.480–1.853	0.864
CHKA expression	High/low	1.920	1.298–2.839	**0.001**	1.867	1.249–2.792	**0.002**
**Disease-specific survival**							
Gender	Male/female	1.057	0.713–1.566	0.783	1.145	0.755–1.735	0.524
Age (years)	≥ 60/< 60	1.233	0.784–1.937	0.364	1.586	0.965–2.606	0.069
Tumor location	Colon / rectum	1.093	0.737–1.622	0.659	1.212	0.780–1.883	0.392
Tumor size (cm)	≥ 5/< 5	1.337	0.890–2.009	0.162	1.258	0.825–1.919	0.287
Differentiation grade	Poor/well + moderate	1.599	1.038–2.461	**0.033**	1.503	0.855–2.642	0.157
Local invasion	pT3-4/pT1-2	2.114	1.100–4.063	**0.025**	1.400	0.706–2.777	0.335
Lymph node metastasis ^b^	Positive /negative	2.384	1.612–3.526	**< 0.001**			
TNM stage	III/I + II	2.384	1.612–3.526	**< 0.001**	2.628	1.322–5.225	**0.006**
Adjuvant chemotherapy	Yes/no	2.017	1.345–3.026	**0.001**	0.808	0.371–1.760	0.591
CHKA expression	High/low	2.394	1.480–3.874	**<0.001**	2.244	1.371–3.675	**0.001**

Abbreviations: HR, hazard ratio; 95% CI, 95% confidence interval.

a Bold type indicates statistical significance.

b Since the stratification of patients by lymph node metastasis (positive / negative) and by TNM stage (III/I + II) was equivalent in the study cohort, the former was not further enrolled into multivariate analysis.

Since there is a differential treatment between early and advanced stages CRC patients, we stratified the CRC cohort according to TNM stage to evaluate the association of CHKA expression with clinical outcomes of patients in different stage subgroups. Stage-based survival analyses demonstrated that high expression of CHKA protein predicted poor DFS and DSS not only in early stage patients (Figure 2C and 2D) but also in advanced stage patients (Figure 2E and 2F).

The independent prognostic value of CHKA expression based on tumor stage was further evaluated with a Cox multivariate regression model. As shown in Table 3, multivariate analysis demonstrated that, for patients with early stage tumors, increased CHKA expression was independently associated with poor DSS (HR = 2.029, 95% CI = 1.046–3.937, *p* = 0.036). For patients with the advanced stage tumors, high CHKA protein expression was independently associated with unfavorable DFS (HR = 2.069, 95% CI = 1.025–4.178, *p* = 0.043) and DSS (HR = 2.412, 95% CI = 1.081–5.382, *p* = 0.032). Taken together, these data suggest that expression level of CHKA could be used as an independent factor for predicting the prognosis of CRC.

**Table 3:** Multivariate analyses of CHKA expression and survival of CRC patients with early or advanced stage tumors

Variables	Categories	Early Stage			Advanced Stage		
		HR	95% CI	*P* value^a^	HR	95% CI	*P* value^a^
**Disease–free survival**							
Gender	Male/female	1.224	0.737–2.033	0.435	0.986	0.569–1.708	0.960
Age (years)	≥ 60/<60	1.489	0.795–2.789	0.214	1.175	0.627–2.202	0.615
Tumor location	Colon/rectum	0.951	0.575–1.571	0.844	1.812	0.997–3.294	0.051
Tumor size (cm)	≥ 5/< 5	0.897	0.552–1.458	0.662	1.862	0.979–3.540	0.058
Differentiation grade	Poor/well + moderate	0.542	0.210–1.397	0.205	1.427	0.781–2.610	0.248
Local invasion	pT3-4/pT1-2	2.048	1.014–4.134	**0.045**	0.669	0.227–1.971	0.466
Adjuvant chemotherapy^b^	Yes/no	1.887	0.843–4.223	0.122			
CHKA expression	High/low	1.561	0.927–2.627	0.094	2.069	1.025–4.178	**0.043**
**Disease–specific survival**							
Gender	Male/female	1.134	0.613–2.100	0.689	1.214	0.684–2.155	0.508
Age (years)	≥ 60/< 60	1.799	0.814–3.975	0.147	1.298	0.656–2.569	0.454
Tumor location	Colon/rectum	0.854	0.469–1.555	0.606	1.771	0.937–3.349	0.079
Tumor size (cm)	≥ 5/< 5	1.098	0.614–1.962	0.753	1.600	0.827–3.097	0.163
Differentiation grade	Poor/well + moderate	0.667	0.212–2.098	0.489	1.548	0.821–2.920	0.177
Local invasion	pT3-4/pT1-2	1.818	0.790–4.184	0.160	0.910	0.274–3.028	0.878
Adjuvant chemotherapy^b^	Yes/no	1.537	0.596–3.964	0.374			
CHKA expression	High/low	2.029	1.046–3.937	**0.036**	2.412	1.081–5.382	**0.032**

Abbreviations: HR, hazard ratio; 95% CI, 95% confidence interval.

a Bold type indicates statistical significance.

b Since all advanced stage (stage III) patients had received adjuvant chemotherapy, adjuvant chemotherapy was not enrolled into the multivariate analysis in this subgroup.

### Combination of CHKA with TNM stage exhibits improved prognostic accuracy for CRC

**Figure 3:**
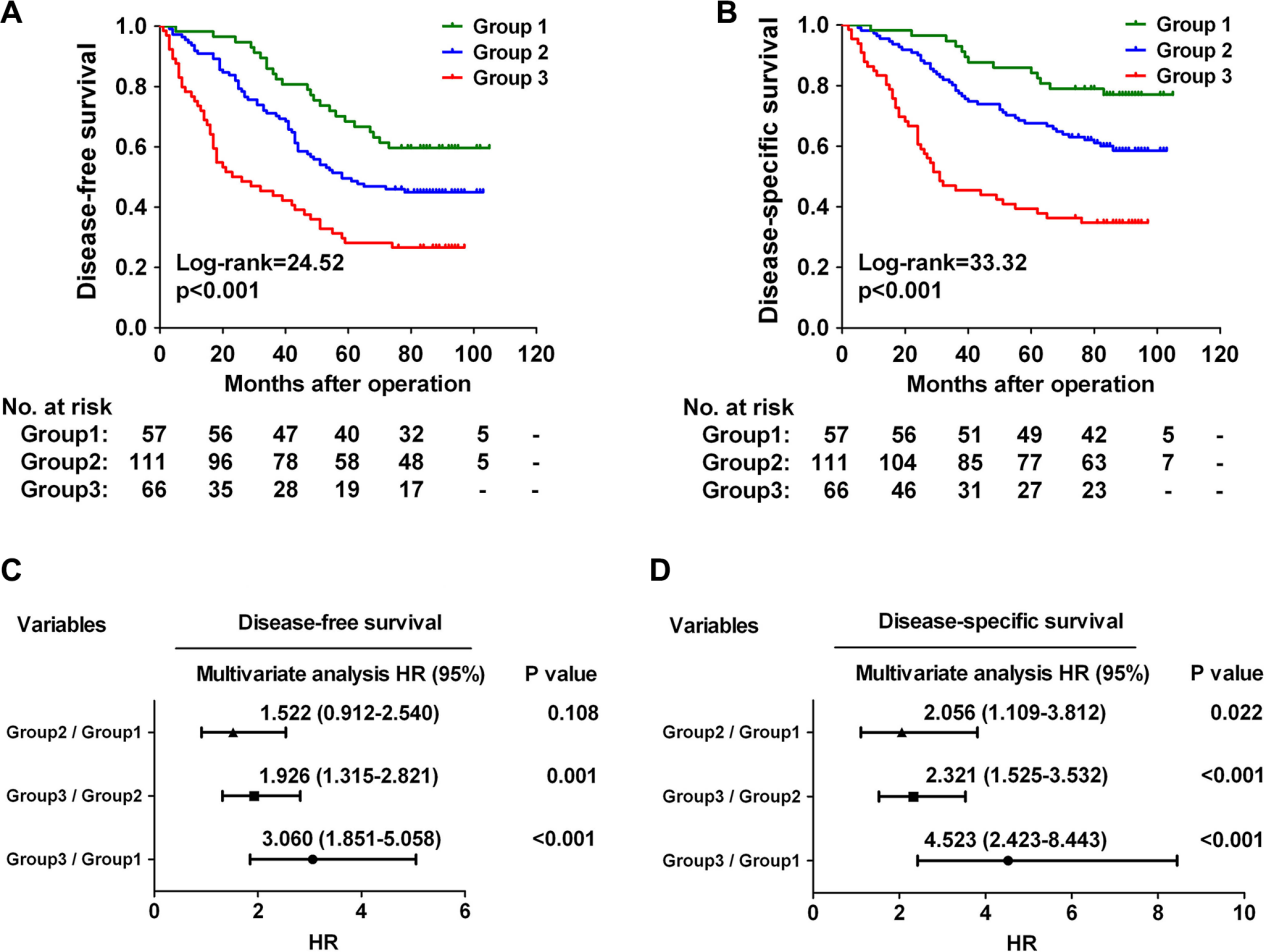
Combination of CHKA with TNM stage exhibits improved prognostic accuracy for CRC (**A–B**) Kaplan-Meier curves for disease-free survival (A) and disease-specific survival (B) of CRC patients in the study cohort according to the three dictinct risk groups: Group 1: both factors low; group 2: one factor high; Group 3: both factors high. The *p*-value was determined using the log-rank test. The absolute number of patients at risk in each subgroup was listed below. (**C–D**) A Cox multivariate analysis of hazard ratios for the disease-free survival (C) and disease-specific survival (D) of CRC patients in the study cohort according to the three different risk groups.

Since both CHKA expression and TNM stage are independent predictors of poor outcome for CRC patients (Table 2), we further evaluated whether the CHKA/TNM stage combination model could provide better prognostic accuracy than single-parameter model. Based on CHKA expression and TNM stage status, we classified the 234 CRC patients into three distinct risk groups: Group 1 (low-risk group, both factors low), low CHKA+early TNM stage; Group 2 (intermediate-risk group, one factor high), either low CHKA+advanced TNM stage or high CHKA+early TNM stage; and Group 3 (high-risk group, both factors high), high CHKA+advanced TNM stage. Survival analysis revealed that patients in Group 3 had the shortest DFS and DSS, while those in Group 1 had the longest DFS and DSS (Figure 3A and 3B, all *p* < 0.001). In addition, Cox multivariate regression analysis demonstrated that patients in Group 3 harbored a 3.060-fold higher risk of cancer recurrence (95% CI = 1.851–5.058, *p* < 0.001) and a 4.523-fold higher risk of death (95% CI = 2.423–8.443, *p* < 0.001) than those in Group 1 (Figure 3C and 3D). Receiver operating characteristic (ROC) curve analysis was used to further compare the prognostic validity of the combined model with CHKA expression alone or TNM stage alone model. The model combining CHKA expression and TNM stage indeed had a better survival predictive power than either CHKA expression alone or TNM stage alone model (Figure S2). Thus, combination of CHKA expression with TNM stage had an increased prognostic value in comparison with either parameter alone.

### Knockdown of CHKA suppresses the proliferation and tumor growth of CRC cells *in vitro* and *in vivo*

**Figure 4:**
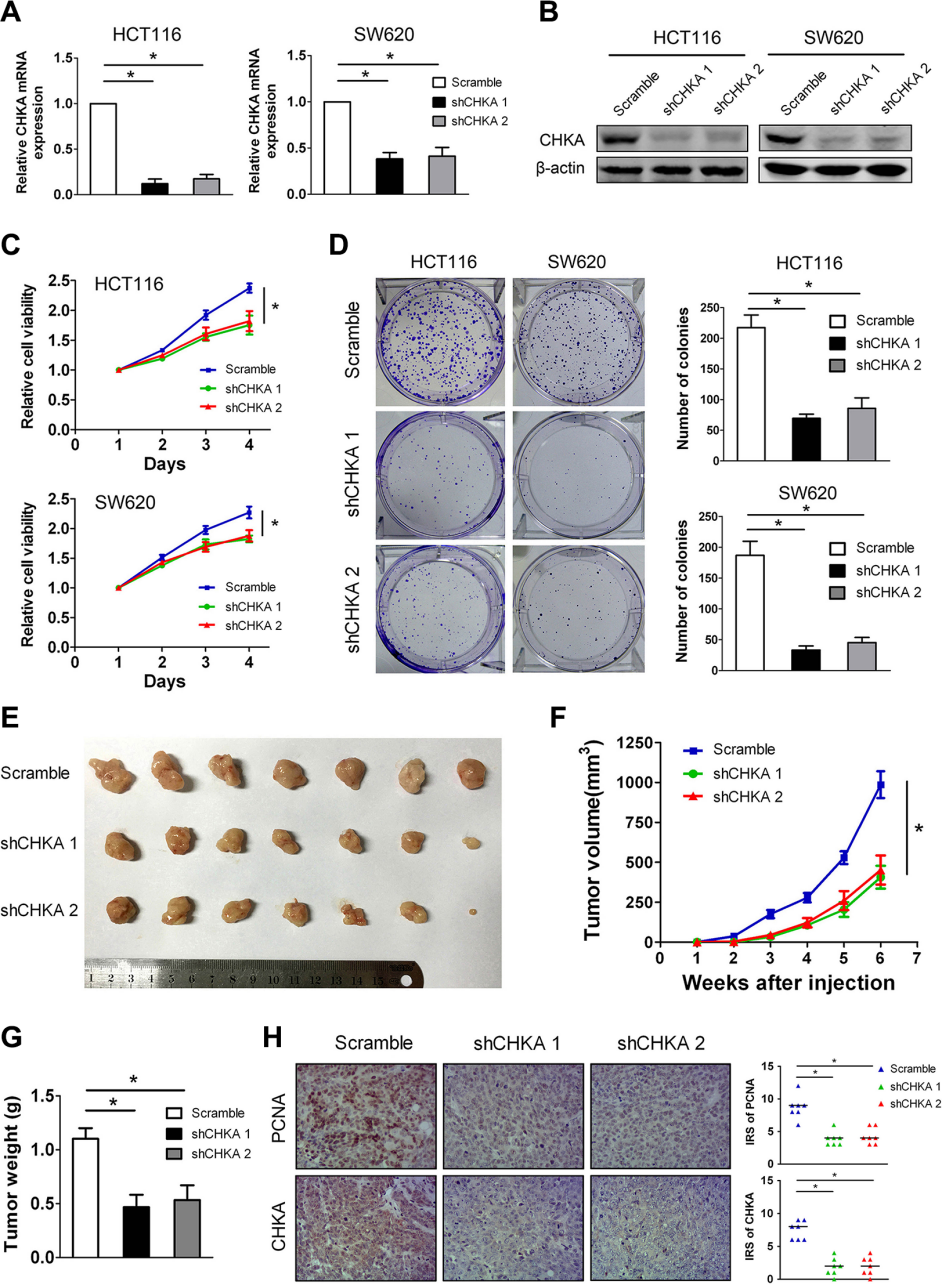
CHKA silencing attenuates the proliferation and tumor growth of CRC cells *in vitro* and *in vivo* (**A–B**) HCT116 or SW620 cells were infected with lentivirus expressing scramble non-targeting shRNA (Scramble) or CHKA shRNA1 (shCHKA 1) or CHKA shRNA2 (shCHKA 2) and the stable knockdown efficiency was confirmed by qPCR (A) and western blot assay (B). (**C**) Cell viability of HCT116 or SW620 cells infected with lentivirus expressing Scramble or shCHKA 1 or shCHKA 2 was determined by the Cell Counting Kit 8 assay. Plots are presented as mean ± SEM of data from three independent experiments. (**D**) Effects of CHKA knockdown on the proliferation of HCT116 or SW620 cells were assessed by the colony-forming assay. Representative results are shown in the left panel. Plots in the right panel are presented as mean ± SEM of data from three independent experiments. (**E**) Xenograft tumor model assays. 2×10^6^ of HCT116 control or CHKA-depleted cells were subcutaneously injected into the lateral flank of nude mice. Subcutaneous xenografts from each group were excised from nude mice (*n* = 7). (**F**) The growth curves of xenografts tumor volumes. (**G**) Tumor weight. Plots are presented as mean ± SEM. (**H**) Indicated xenograft tumors were subjected to immunohistochemical staining of PCNA and CHKA. Representative images are shown in the left panel (Magnification,×200). Median IRS values of PCNA and CHKA staining in the xenograft tumor samples are shown in the right panel. *p < 0.05.

Given that increased CHKA expression in CRC is a common molecular incident and correlated with aggressive tumor characteristics, we hypothesize that depletion of CHKA can exert inhibitory effects on CRC development and progression. To test the hypothesis, we constructed stable CHKA-depleted cell models in two human CRC cell lines, HCT116 and SW620, which have high levels of endogenous CHKA, using two lentivirus-mediated shRNAs targeting CHKA. The efficacy of knockdown of CHKA expression was confirmed by qPCR and western blot analysis (Figure 4A and 4B). The Cell Counting Kit-8 assay showed that depletion of CHKA resulted in a significant decrease of the proliferation rate in both HCT116 and SW620 cells (Figure 4C). In addition, CHKA downregulation greatly impaired the colony-formation ability in each cell line (Figure 4D).

To verify the *in vivo* consequences of CHKA knockdown, HCT116 control or CHKA-depleted cells were injected subcutaneously into the dorsal flank of nude mice and tumor growth was monitored. As shown in Figure 4E and 4F, mice injected with CHKA-depleted cells showed significantly reduced xenograft tumor growth compared with those injected with control cells. The average tumor weight was also significantly decreased in the CHKA depletion groups compared with the control group (Figure 4G). In addition, the xenograft tumors were subjected to immunohistochemical staining of proliferating cell nuclear antigen (PCNA), which is used as a proliferation marker. Notably, the number of PCNA-positive nuclei was markedly decreased in the CHKA-depleted xenograft tumors when compared to the control counterparts (Figure 4H). These results indicate that CHKA plays a positive role in CRC proliferation.

### Depletion of CHKA impairs the invasion and metastasis of CRC cells *in vitro* and *in vivo*

**Figure 5:**
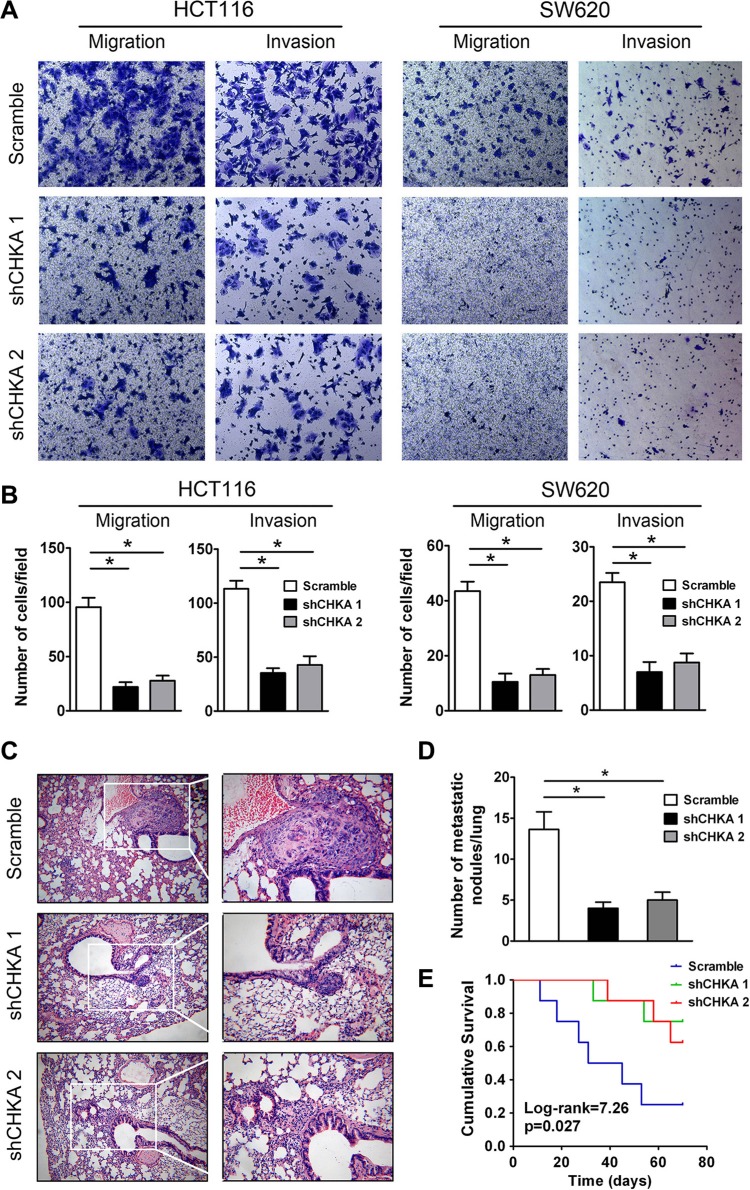
Depletion of CHKA suppresses the invasive and metastatic potential of CRC cells *in vitro* and *in vivo* (**A**) Effects of CHKA knockdown on the migration and invasion of HCT116 or SW620 cells were determined by the transwell migration assay and matrigel invasion assay, respectively. Representative results are shown. (**B**) Plots for panel A are presented as mean ± SEM of data from three independent experiments. (**C**) Lung metastasis tumor model assays. 1 × 10^6^ of HCT116 control or CHKA-depleted cells were injected into the tail vein of nude mice (*n* = 8). Ten weeks post inoculation, mice were sacrificed and metastatic tumor colonies in the lung were examined microscopically. Representative images of H&E staining of lung metastatic nodules in each group are shown. (Magnification, left panel, ×100; right panel, ×200) (**D**) The number of metastatic nodules in the lungs of each group is presented as mean ± SEM. (**E**) Kaplan-Meier curves for overall survival of mice in each group. The *p*-value was determined using the log-rank test. *p < 0.05.

To determine the role of CHKA in the motility of CRC cells, transwell migration and matrigel invasion assays were performed in CHKA-depleted and control cells. The transwell migration assay demonstrated that silencing endogenous CHKA expression markedly suppressed the migratory capabilities of HCT116 and SW620 cells. Consistently, CHKA-silenced cells displayed a significantly reduced invasive potential through matrigel as compared with their control cells (Figure 5A and 5B).

We further evaluated the effects of CHKA downregulation on metastasis of CRC cells *in vivo* using an experimental lung metastasis model. HCT116 control or CHKA-silenced cells were injected into the lateral tail vein of nude mice to induce lung metastasis. Ten weeks later, fewer and smaller micrometastatic lesions were detected microscopically in the lungs of mice inoculated with CHKA-silenced cells than in those of mice inoculated with control cells (Figure 5C and 5D). Moreover, mice injected with CHKA-depleted cells had a significantly longer survival time compared with those injected with control cells (Figure 5E). Collectively, these findings suggest that CHKA is essential for the invasive and metastatic potential of CRC cells both *in vitro* and *in vivo*.

### EGFR/PI3K/AKT pathway plays a critical role in mediating CHKA function

**Figure 6:**
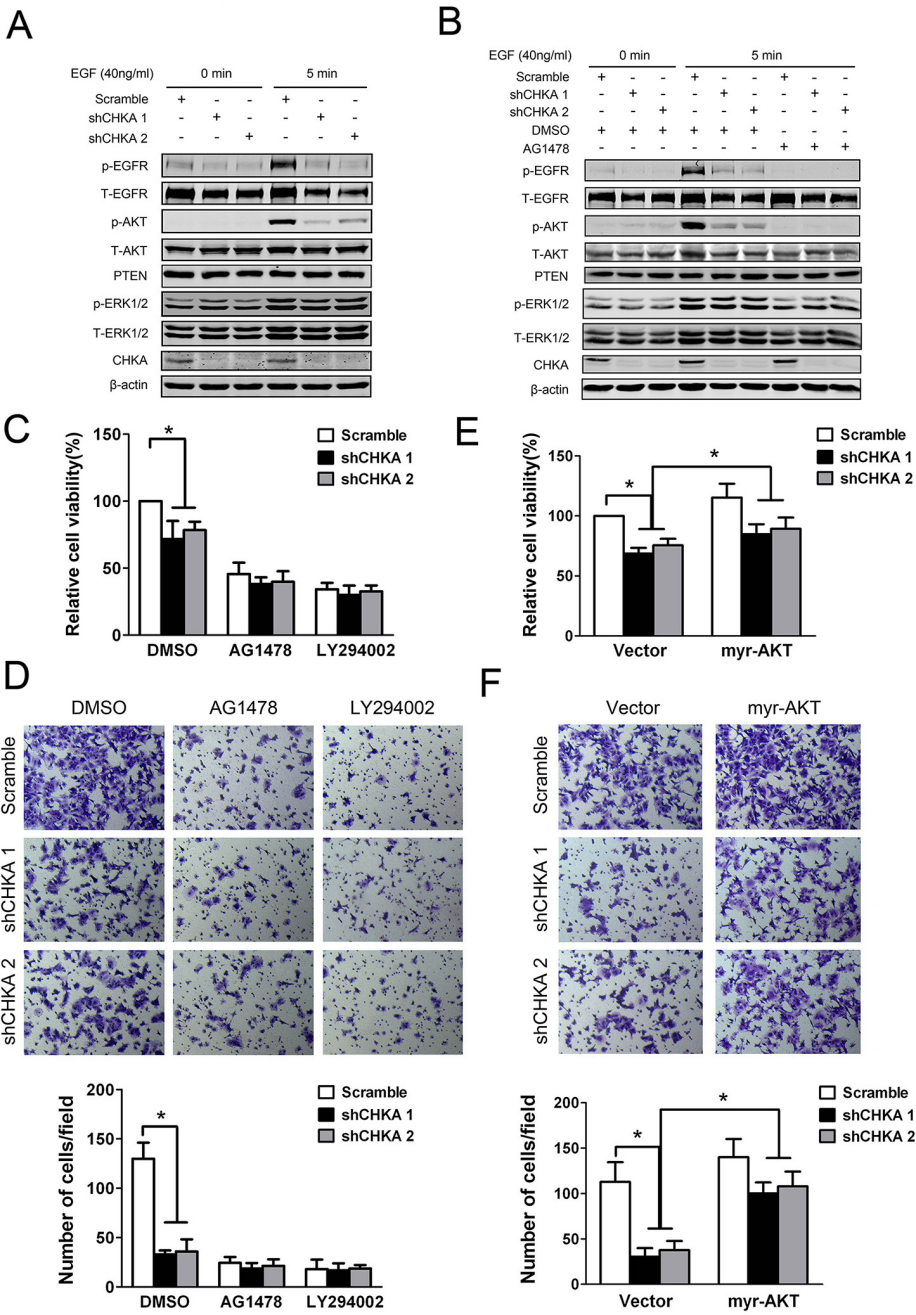
EGFR/PI3K/AKT pathway is involved in mediating CHKA action (**A**) HCT116 control or CHKA-depleted cells were serum starved overnight followed by treated with or without EGF (40 ng/mL) for 5 minutes and then cell lysates were harvested and subjected to western blot assay and probed with the indicated antibodies. (**B**) Indicated cells were serum starved overnight followed by pre-incubated with DMSO or AG1478 (100 nM) for 2 hr and then treated with or without EGF (40 ng/mL) for 5 minutes. Cell lysates were harvested and subjected to western blot assay and probed with the indicated antibodies. (**C**) Indicated cells were pre-incubated with DMSO or AG1478 (100 nM) or LY294002 (10 μM) for 2 hr and the cell viability was measured by Cell Counting Kit 8 assay at 72 hr. Data are given as percentage of scramble lentivirus-infected DMSO-treated HCT116 cells as control which was set at 100%. Plots are presented as mean ± SEM of data from three independent experiments. (**D**) Indicated cells were pre-incubated with DMSO or AG1478 (100 nM) or LY294002 (10 μM) for 2 hr and then subjected to matrigel invasion assays. Representative results are shown. Plots in the lower panel are presented as mean ± SEM of data from three independent experiments. (**E**) Indicated cells were transiently transfected with control vector or myr-AKT (the constitutively active form of AKT) plasmid and the cell viability was measured by Cell Counting Kit 8 assay at 72 hr. Data are given as percentage of scramble lentivirus-infected control vector-transfected HCT116 cells as control which was set at 100%. Plots are presented as mean ± SEM of data from three independent experiments. (**F**) Indicated cells were transiently transfected with control vector or myr-AKT plasmid. Twenty four hours post transfection, cells were subjected to matrigel invasion assays. Representative results are shown. Plots in the lower panel are presented as mean ± SEM of data from three independent experiments. *p < 0.05.

We next sought to explore the signaling mechanisms responsible for mediating the effects of CHKA knockdown on cell growth and motility. Interestingly, activation of EGFR was markedly suppressed in CHKA-silenced cells upon EGF stimulation (Figure 6A). Meanwhile, phosphorylation levels of AKT were also significantly decreased in CHKA-silenced cells compared with control cells in response to EGF stimulation. While no substantial changes were observed in the levels of PTEN or ERK1/2 phosphorylation between CHKA-depleted and control cells. In addition, pretreatment of cells with an EGFR-specific tyrosine kinase inhibitor (AG1478) not only effectively blocked the activity of EGFR but also eliminated the distinct activation of AKT signaling between CHKA-depleted and control cells, indicating that CHKA-mediated AKT activation occurs mainly downstream of EGFR activation (Figure 6B).

Importantly, pretreatment of cells with AG1478 as well as LY294002, a specific inhibitor of PI3K, profoundly suppressed the proliferative and invasive potentials of HCT116 cells and diminished the differences in proliferation and invasion between CHKA-depleted and control cells (Figure 6C and 6D). To further determine whether the impaired AKT signaling is required for CHKA depletion-mediated inhibition of CRC cell growth and invasion, HCT116 cells were transiently transfected with control vector or myr-AKT (the constitutively active form of AKT) plasmid. As shown in Figure 6E and 6F, activation of AKT signaling by ectopic expression of myr-AKT significantly reversed the reduced tumor cell proliferative and invasive capabilities caused by CHKA knockdown. These results reveal a critical role for EGFR/PI3K/AKT pathway in CHKA-facilitated growth and invasiveness of CRC cells. Consistently, immunohistochemical analysis revealed a positive correlation between CHKA expression and phospho-AKT levels in human CRC tissue samples (Figure S3).

## DISCUSSION

CRC is still one of the most dreadful human malignant diseases due to tumor recurrence and metastasis after surgical resection. Therefore, a better understanding of the molecular mechanisms underlying the pathogenesis of the disease is warranted in order to identify biomarkers for prediction and intervention. In the present study, we reported that CHKA protein expression was upregulated in CRC tissues and CRC-derived cell lines and was significantly correlated with several important clinicopathologic parameters including lymph node metastasis, tumor stage, disease recurrence and vital status. Higher expression of intratumoral CHKA protein predicted poorer DFS and DSS and was an independent unfavorable prognostic indicator for CRC patients. Combination of CHKA with TNM stage had better prognostic accuracy than either parameter alone. In addition, CHKA knockdown markedly suppressed CRC growth and metastasis both *in vitro* and *in vivo*. We further demonstrated that EGFR/PI3K/AKT pathway was essential for mediating CHKA action.

Using qPCR and western blot analysis, we demonstrated that CHKA expression was frequently upregulated in CRC tissue samples at both the mRNA and protein levels. Increased protein expression of CHKA in CRC tissues has been reported previously by Ramirez de Molina *et al*. [10]. Our western blot data were consistent with their findings. Moreover, immunohistochemical analysis of 234 paired paraffin-embedded CRC specimens revealed that immunostaining of CHKA protein was observed mainly in the cytoplasm and 70.9% (166/234) of the cancerous tissues were classified as CHKA-positive, whereas only 15.8% (37/234) of the adjacent normal mucosa tissues showed positive CHKA immunoreactivity. Consistently, both mRNA and protein levels of CHKA were markedly increased in the CRC-derived cell lines examined relative to the normal colon epithelial cell line NCM460, which was also in agreement with a recent observation [35]. In addition, although CRC cell lines are enriched with DNA mismatch repair deficient ones, increased expression of CHKA in CRC cell lines seems to be irrespective of their mismatch repair status. Thus, our results definitely confirmed the significant upregulation of CHKA protein expression in CRC.

Interestingly, analyzing the association between intratumoral CHKA expression and clinicopathologic features revealed a significant positive correlation of CHKA expression with lymph node metastasis, TNM stage and disease recurrence, which are all hallmarks for poor prognosis of CRC [2]. Further analysis confirmed that expression levels of CHKA were significantly higher in advanced stage CRCs than in early stage CRCs, suggesting that CHKA is highly expressed in a subset of human CRCs which shows a more aggressive behavior of the disease. Importantly, survival analyses of the complete study cohort demonstrated that patients with higher CHKA expression had significant shortened DFS and DSS than those with lower CHKA expression. In addition, CHKA protein emerged as an independent predictor of recurrence and survival along with TNM stage. Up to now, the expression of CHKA and its prognostic significance have been demonstrated in lung and liver cancers [33, 34]. However, its prognostic implication has not been assessed in CRC. Thus, the current study is the first to report the prognostic value of CHKA expression in CRC. Our results were similar to previous findings in NSCLC and HCC. While, Challapalli *et al.* recently showed that increased CHKA expression was associated with a trend towards poor progression-free survival but had no correlation with overall survival of patients with prostate cancer [36]. The different results on the prognostic value of CHKA in different malignances indicate that its prognostic significance is tissue-dependent and varies with the type of malignancy. Of note, our results indicated that TNM stage also is an important prognostic factor in CRC (Figure S1), which is in keeping with its well established adverse prognostic effect [37] and proves that our cohort was representative and that the survival analyses were valid. Moreover, stage-based survival analyses revealed that higher CHKA expression maintained its prognostic value in predicting poorer DFS and DSS in patients with different stage tumors. These findings should be of particular interest especially for patients with early stage tumors. Generally speaking, patients who had early stage tumors have a relatively favorable prognosis than those who had advanced stage tumors. However, a subgroup of early stage patients have an increased risk of early recurrence and death. Therefore, identification of this high-risk subgroup of early stage patients by markers is of great clinical need for appropriate treatment. Together with the finding that the CHKA/TNM stage combination had a more powerful efficiency for predicting the outcome of patients than either parameter alone, our current results suggest that combination of CHKA with TNM stage may serve as a promising prognostic biomarker to stratify CRC patients into distinct risk subgroups and guide individualized therapy.

The significant correlations between CHKA expression in tumors and aggressive clinical behaviors and poor prognosis of CRC patients prompted us to investigate whether CHKA plays a functional role in CRC progression and dissemination. Indeed, both our *in vitro* and *in vivo* data clearly demonstrated that CHKA silencing hampered the proliferation, invasion and metastasis of CRC cells. Although the pro-growth and invasive phenotype of this oncoprotein has been well documented in a wide spectrum of human cancers [12, 14, 19, 22, 38, 39], the present work, to our knowledge, provides the first evidence that CHKA expression is critical for CRC invasion and metastasis in addition to tumor growth, which may explain the observed association between increased CHKA expression and aggressive clinical phenotypes. Our results also suggest that CHKA may represent a promising target for therapeutic intervention against invasive and metastatic CRC. Moreover, our data provide additional evidence to what has been reported by other groups using either specific siRNAs or small molecule inhibitors towards CHKA. Knockdown of CHKA by RNAi has been demonstrated to induce differentiation and reduce proliferation [12, 40], prevent mitotic entry [8], selectively trigger cancer cell apoptosis [41], suppress migration and invasion [21, 22, 31], and sensitize cancer cells to chemotherapeutics [22, 42]. Consistently, pharmacological inhibitors of CHKA has also displayed antiproliferative, proapoptotic and antitumoral effects against multiple tumor-derived cancer cells as well as tumor xenografts [18, 23, 32, 43–46]. Indeed, one CHKA inhibitor, designated as TCD-717 or RSM-932A, has recently completed Phase I clinical trials for the treatment of advanced solid tumors (ClinicalTrial.gov Identifier: NCT01215864) [27]. Thus, together our findings with what has been reported so far, inhibition of CHKA may have a broad spectrum application as an attractive novel anticancer strategy.

EGFR and PI3K/AKT signalings have been implicated in tumorigenesis, invasion and metastasis of cancer including CRC [47, 48]. Interestingly, we found that knockdown of CHKA markedly suppressed the phosphorylation levels of EGFR and AKT upon EGF stimulation and that the CHKA-dependent activation of AKT was mainly mediated through EGFR. This observation prompted us to further determine whether EGFR/PI3K/AKT signaling is involved in mediating the oncogenic property of CHKA in CRC. As expected, blockade of the activation of EGFR as well as PI3K/AKT signaling by their respective pharmacological inhibitors eliminated the discrepant capacities in proliferation and invasion between control and CHKA-silenced cells; conversely, activation of AKT signaling by ectopic expression of myr-AKT effectively reversed the inhibitory effects of CHKA knockdown on CRC cell proliferation and invasion. Moreover, a positive correlation between CHKA expression and phospho-AKT levels was observed in clinical CRC samples, which further supports the activation of AKT by CHKA in human CRCs. Thus, our data reveal that CHKA facilitates CRC progression, at least in part, through EGFR/PI3K/AKT-dependent pathway. These results are in accordance with the reported critical role of this signaling cascade in the pathogenesis of diverse cancer types [49–52], and also supported by the recent uncovered associations between CHKA and EGFR and PI3K/AKT pathway in other tissues. Miyake *et al*. demonstrated that CHKA binds to EGFR to form a complex in a c-Src-dependent manner, thereby contributing to the regulation of breast cancer cell proliferation and tumorigenesis [53]. George *et al.* found CHKA downregulation attenuates phosphorylation of EGFR stimulated by angiotensin II in human mammary epithelial cells, identifying it as one of the genes involved in angiotensin II-mediated EGFR transactivation process [54]. In addition, it has been clarified that CHKA positively regulates AKT phosphorylation at the Ser473 residue and is required for the activation of AKT in breast carcinoma cells [55] and that genetic silencing and pharmacological inhibition of CHKA suppresses both PI3K/AKT and MAPK signaling in HeLa cells and T-lymphoma cells [18, 25, 26]. Taken together, these studies indicate that CHKA activity may be essential for tumor progression and also for the activation of downstream oncogenic signaling pathways.

Of note, unlike the findings shown in other cell systems [18, 25, 26], we did not observe a significant inhibitory effect on ERK signaling following CHKA knockdown in the CRC cell lines used here. Recently, the inability of CHKA silencing to attenuate MAPK and PI3K/AKT signaling has been observed in ovarian cancer cells [22, 31]. Thus, the discrepant results on the signaling pathways affected by CHKA knockdown reported by different groups may be attributed to the differences in cancer types studied, cellular systems used, or siRNA molecules employed and further investigation is needed to define the basis for these discrepancies.

More recently, Asim *et al.* provided direct evidence that CHKA acts as a molecular chaperone for androgen receptor, enhancing its stability and function [21]. Their discovery not only offers one explanation for the effect of CHKA knockdown in prostate cancer, but also supports the importance of a non-catalytic scaffolding function of CHKA protein, rather than or in addition to its catalytic activity, in promoting cancer cell survival, which has also been demonstrated recently in other epithelial malignancies [53, 56]. Although in this study we did not observe a significant effect of CHKA knockdown on EGFR distribution or c-Src levels in HCT116 cells (data no shown), it would be interesting to examine whether CHKA could interact with EGFR in CRC, and if so whether this interaction is also dependent on c-Src activity. Additionally, given the complex of AKT signaling pathways, future work should be warranted to investigate whether other upstream regulators are involved in CHKA-mediated AKT activation.

The current study had several limitations. First, information on several commonly studied tumoral molecular events, including oncogenic mutations of KRAS, BRAF and PIK3CA, and DNA microsatellite status, was not available for analysis. Hence, the possible associations between CHKA expression and these known molecular prognostic markers have yet to be determined. Besides, only HCT116 cells, which are microsatellite-instable CRC cells, have been employed for *in vivo* functional experiments. Thus, our *in vivo* data support the increased local invasive and metastogenic properties conferred by CHKA expression to microsatellite-instable CRC. Whether CHKA has a similar role in microsatellite-stable CRC requires further verification. In addition, the mechanism by which CHKA activates EGFR/PI3K/AKT pathway and contributes to the pathogenesis and progression of CRC remains to be elucidated. Further studies are needed to validate the robustness of our findings before clinical translation and provide a better insight into the molecular events involved in the CHKA-facilitated cancer progression and metastasis.

In summary, we report here, for the first time, that upregulated CHKA expression correlates with disease progression and unfavorable postoperative prognosis of patients with CRC. CHKA plays a key role in CRC proliferation and metastasis and could be a useful prognostic biomarker for this malignancy. Combination of CHKA with TNM stage or other parameters may enhance its performance in prognostic prediction. In addition to its prognostic value, our findings provide additional rationale for the potential utility of CHKA-targeted therapy in the treatment of CRC.

## MATERIALS AND METHODS

### Patients and specimens

Formalin-fixed paraffin-embedded tissue specimens from 234 stages I–III CRC patients who received curative surgery in the 150th Hospital of PLA from June 2006 to March 2009 were retrieved for immunohistochemistry. The study cohort consisted of CRC patients with typical adenocarcinoma histology as confirmed by pathological analysis. Detailed clinicopathologic characteristics of the 234 patients were listed in Table 1. The median age at time of diagnosis was 65 years (range, 24–91 years). The follow-up period was defined as the interval from the date of surgery to the date of death or last follow-up. The median follow-up time of the study cohort was 82.5 months (range, 2–105 months) and the latest follow-up was updated in March 2015. Patients alive at the end of follow-up were censored. Disease-specific survival (DSS) was defined as the interval from the date of surgery to the date that patient died of CRC. Disease-free survival (DFS) was defined as the interval from the date of surgery to the date of disease recurrence; if recurrence was not diagnosed, patients were censored on the date of death or last follow-up. Patients were excluded from the study cohorts with the following exclusion criteria: previously received any anticancer therapy; impaired heart, lung, liver, or kidney function; previous malignant disease; and died from other causes. Tumor stage was classified according to the 7th Edition tumor-node-metastasis (TNM) classification of the American Joint Committee on Cancer Staging. 5-fluoruracil-based adjuvant chemotherapy was administered to all stage III patients and a subgroup of stage II patients who had at least one of the following risk factors: pT4 or poorly differentiated tumors discovered after surgery.

Fresh-frozen CRC samples obtained from an independent set of 63 stages I–III primary CRC patients who received curative surgery in the 150th Hospital of PLA from April 2013 to Oct 2013 were used for qPCR and western blot analysis. Detailed clinicopathologic characteristics of the 63 patients were listed in Table S4.

Written informed consent was obtained from each patient and this study was conducted in accordance with the ethical standards and according to the Declaration of Helsinki and approved by the institutional Ethics Committee of the 150th Hospital of PLA.

### Real-time qPCR analysis

Real-Time qPCR was performed as described previously [57]. Briefly, total RNAs were isolated from frozen specimens or cell lines using TRIzol Reagent (Invitrogen). Reverse transcription (RT) was performed using RevertAid^TM^ First Strand cDNA Synthesis Kit (Fermentas) according to the manufacturer's instructions. After the RT reaction, the cDNA template was quantitated using real-time PCR technology. PCR was performed on ABI Prism 7500 Sequence Detection System with SYBR Premix Ex Taq^TM^ II (Takara) using the 2^−ΔΔCT^method. Gene expression results were normalized by internal control β-actin. The primers used in this study are as follows: CHKA (NM_001277.2) forward, 5′-TGGTCCATTGTACAAGCCAA-3′; reverse, 5′-CAAGCTTCCTCTTCTGGTGG-3′; β-actin forward, 5′-AATCGTGCGTGACATTAAGGAG-3′; reverse, 5′-ACTGTGTTGG CGTACAGGTCTT-3′. Each sample was tested in duplicate.

### Western blot analysis

Western blot assay was performed as described previously [58]. Briefly, tumor specimens or whole-cell extracts were prepared in lysis buffer [Tris-HCl (20 mM), pH 7.4, NaCl (150 mM), glycerol (10%), Nonidet P-40 (0.2%), EDTA (1 mM), EGTA (1 mM), PMSF (1 mM), NaF (10 mM), aprotinin (5mg/ml), leupeptin (20 mM), and sodium orthovanadate (1 mM)] and centrifuged at 12,000 g for 30 min. Protein concentrations were measured using the BCA assay. Immunoblotting was performed using specific primary antibodies and immunocomplexes were incubated with appropriate horseradish peroxidase-conjugated or fluorescein-conjugated secondary antibodies and then detected using an ECL kit (Santa Cruz Biotechnology) or an Odyssey fluorescence scanner (Li-Cor, Gene Company). β-actin was used as a loading control. The primary antibodies specific for CHKA, phospho-EGFR (p-EGFR), p-AKT, p-ERK1/2, total-EGFR (T-EGFR), T-AKT, T-ERK1/2 and PTEN were purchased from Cell Signaling Technology. anti-β-actin antibody was from Santa Cruz Biotechnology.

### Immunohistochemistry

Immunohistochemistry of paraffin-embedded tissue sections was performed as described previously [59]. Briefly, sections were deparaffinized and rehydrated. The endogenous peroxidase activity was blocked with 3% H_2_O_2_ for 10 minutes. Antigens were retrieved with citrate buffer (10 mM, pH 6.0) for 15 minutes at 100°C in a microwave oven. After blocking, the sections were incubated with a primary anti-CHKA antibody (Sigma-Aldrich, HPA024153, 1:50) or a primary anti-PCNA antibody (Abcam, ab92552, 1:200) at 4°C overnight in a moist chamber followed by incubated with an anti-rabbit peroxidase-conjugated secondary antibody (Santa Cruz) at room temperature for 30 minutes. Finally, the visualization signal was developed with diaminobenzidine (Dako) and the slides were counterstained with hematoxylin.

Stained sections were evaluated in a blinded manner without prior knowledge of the clinical data using the German immunoreactive score (IRS) as described previously [60]. Briefly, staining intensity was graded as “0” (negative), “1” (weak), “2” (moderate) and “3” (strong); staining extent was graded as “0” (<5%), “1” (5–25%), “2” (25–50%), “3” (50–75%) or “4” (>75%). The scores of the staining intensity and the staining extent were multiplied to give a final IRS of 0–12, and the median IRS value (IRS = 3) of intratumoral CHKA expression was chosen as the optimal cut-off for differentiating between final high and low CHKA expression levels based on the ROC curve analysis (Sensitivity 66.2%, Specificity 93.2%). An IRS of ≥ 3 was used to define tumors with high CHKA expression and an IRS of < 3 was used to indicate tumors with low CHKA expression. Discrepancies in the IRS were resolved by discussing together with other pathologists to reach a consensus.

### Reagents

Dimethyl sulfoxide (DMSO), crystal violet, the EGFR tyrosine kinase inhibitor AG1478 and the PI3K specific inhibitor LY294002 were all purchased from Sigma-Aldrich.

### Cell lines and cell culture

Normal human colon mucosal epithelial cell line NCM460 and CRC cell lines LS174T, DLD1, HT29, HCT116, SW480, and SW620 were purchased from Cell Bank of Type Culture Collection of Chinese Academy of Sciences (Shanghai, China). All cell lines were maintained at 37°C in a humidified incubator containing 5% CO_2_ in Dulbecco's modified Eagle's medium (DMEM) or RPMI-1640 supplemented with 10% heat-inactivated fetal bovine serum and passed every 2–3 days to maintain logarithmic growth.

### Lentivirus infection and transient transfection

Lentiviral vectors containing human CHKA short-hairpin RNA (shCHKA 1 or shCHKA 2) or scrambled non-targeting shRNA (Scramble) were prepared by the Genechem Company (Shanghai, China). The following shRNAs specific for CHKA were used: shRNA1: 5′-GACTGTGGTCCATTGTACAAGCCAA-3′; shRNA2: 5′-CATGCTGTTCCAGTGCTCC-3′. A scramble non-targeting shRNA was used as control. Lentivirus infection experiments were performed as described previously [61]. Briefly, cells were infected with the indicated virus at a multiplicity of infection (MOI) of 10 in the presence of polybrene (8 mg/mL) for 8 hr. Twenty four hours later, the supernatant was replaced with fresh medium. After infection, stable colonies were selected in medium containing 3 μg/mL puromycin for 2–3 weeks. Expression of CHKA in the infected cells was validated by qPCR and western blot assay. For plasmid transfection experiments, cells were transiently transfected using PEI (Polyplus; AFAQ) as described previously [59] and expression of AKT in the transfected cells was validated by western blot assay (Figure S4).

### Cell proliferation assay

The cell proliferation assay was performed using the Cell Counting Kit-8 solution (Dojindo Laboratories) according to the manufacturer's instruction. Briefly, cells were seeded at a density of 4 × 10^3^/well in 96-well plates and treated with 10 μL/well of the Cell Counting Kit-8 solution and cell viability was measured at the indicated times. The optical density of the well was measured at 450 nm using a microplate reader.

### Colony-formation assay

Cells were trypsinized to generate a single-cell suspension, and 500 cells/well were seeded into 6-well plates. Dishes were returned to the incubator for 14 days, and the colonies were fixed with methanol for 1 hr at room temperature and then stained with 0.5% crystal violet for additional 1 hr.

### Cell migration and invasion assay

Migration and invasion assays were performed as described previously [59]. Briefly, Cells were trypsinized, centrifuged, and resuspended in serum-free medium followed by plated into the upper chamber at a density of 2 × 10^5^/well. Complete medium (700 μL) was added to the lower chamber as a chemoattractant. After incubation for 16–18 hr for the migration assay, or after incubation for 20–24 hr for the invasion assay, cells were fixed in methanol and stained with 0.1% crystal violet. Cells on the upper surface of the chamber were removed by wiping with a cotton swab and migration and invasion was determined by counting the cells that migrated to the lower side of the chamber using a microscope at × 100 magnification. Six random microscopic fields were counted per chamber in each group, and these experiments were repeated at least three times.

### *In vivo* xenograft and metastasis tumor assays

Xenograft tumor model was performed as described previously [59]. Briefly, six-week-old nude mice, purchased from the Animal Center of the Second Military Medical University, were subcutaneously injected with the 2 × 10^6^ indicated cells into the lateral flanks of each mouse. Tumor development was observed weekly with a caliper, and the tumor volume was calculated using the following formula: larger diameter × (smaller diameter)^2^/2. For lung metastatic model, nude mice were injected with 1 × 10^6^ indicated cells through the tail vein. Mice were sacrificed at ten weeks post injection. The lungs of each mouse were separated and fixed for H&E staining and lung metastatic foci were detected under microscope. All animals were housed in cages under standard conditions and animal work was conducted according to national and international guidelines and was approved by the Second Military Medical University Animal Care Facility.

### Statistical analysis

Data were presented as mean ± standard error of the mean (SEM) unless otherwise indicated. Pearson chi-square test or Fisher exact test was used to analyze the relationship between CHKA expression and clinicopathologic features. Mann-Whitney *U* test was used to compare CHKA levels between groups. Kaplan-Meier analysis with log-rank test was used to assess patients' survival between subgroups. Cox proportional hazards regression model was applied for the univariate and multivariate analysis of the effect of each variable on survival. The statistical significance of differences was determined using the One-way ANOVA in multiple groups, with the Tukey-Kramer multiple comparison test for post-hoc comparisons. A prognostic combination model was constructed using the significant variables from the Cox multivariate analysis, and ROC curve analysis was performed to compare the sensitivity and specificity for the prediction of survival as described previously [62–64]. All statistical analyses were carried out using SPSS PASW Statistics 18.0 software (SPSS, Inc., Chicago, IL), and *p* value < 0.05 was considered to be statistically significant.

## SUPPLEMENTARY MATERIAL FIGURES AND TABLES



## References

[1] Torre LA, Bray F, Siegel RL, Ferlay J, Lortet-Tieulent J, Jemal A (2015). Global cancer statistics, 2012. CA Cancer J Clin.

[2] Brenner H, Kloor M, Pox CP (2014). Colorectal cancer. Lancet.

[3] Guo P, Huang ZL, Yu P, Li K (2012). Trends in cancer mortality in China: an update. Ann Oncol.

[4] Glunde K, Bhujwalla ZM, Ronen SM (2011). Choline metabolism in malignant transformation. Nat Rev Cancer.

[5] Aoyama C, Liao H, Ishidate K (2004). Structure and function of choline kinase isoforms in mammalian cells. Prog Lipid Res.

[6] Wu G, Vance DE (2010). Choline kinase and its function. Biochem Cell Biol.

[7] Lacal JC (2015). Choline kinase as a precision medicine target for therapy in cancer, autoimmune diseases and malaria. Precis Med.

[8] Gruber J, See Too WC, Wong MT, Lavie A, McSorley T, Konrad M (2012). Balance of human choline kinase isoforms is critical for cell cycle regulation: implications for the development of choline kinase-targeted cancer therapy. FEBS J.

[9] Nakagami K, Uchida T, Ohwada S, Koibuchi Y, Suda Y, Sekine T, Morishita Y (1999). Increased choline kinase activity and elevated phosphocholine levels in human colon cancer. Jpn J Cancer Res.

[10] Ramirez de Molina A, Rodriguez-Gonzalez A, Gutierrez R, Martinez-Pineiro L, Sanchez J, Bonilla F, Rosell R, Lacal J (2002). Overexpression of choline kinase is a frequent feature in human tumor-derived cell lines and in lung, prostate, and colorectal human cancers. Biochem Biophys Res Commun.

[11] Ramirez de Molina A, Gutierrez R, Ramos MA, Silva JM, Silva J, Bonilla F, Sanchez JJ, Lacal JC (2002). Increased choline kinase activity in human breast carcinomas: clinical evidence for a potential novel antitumor strategy. Oncogene.

[12] Glunde K, Raman V, Mori N, Bhujwalla ZM (2005). RNA interference-mediated choline kinase suppression in breast cancer cells induces differentiation and reduces proliferation. Cancer Res.

[13] Iorio E, Mezzanzanica D, Alberti P, Spadaro F, Ramoni C, D'Ascenzo S, Millimaggi D, Pavan A, Dolo V, Canevari S, Podo F (2005). Alterations of choline phospholipid metabolism in ovarian tumor progression. Cancer Res.

[14] Hernando E, Sarmentero-Estrada J, Koppie T, Belda-Iniesta C, Ramírez de Molina V, Cejas P, Ozu C, Le C, Sánchez JJ, González-Barón M, Koutcher J, Cordón-Cardó C, Bochner BH (2009). A critical role for choline kinase-alpha in the aggressiveness of bladder carcinomas. Oncogene.

[15] Iorio E, Ricci A, Bagnoli M, Pisanu ME, Castellano G, Di Vito M, Venturini E, Glunde K, Bhujwalla ZM, Mezzanzanica D, Canevari S, Podo F (2010). Activation of phosphatidylcholine cycle enzymes in human epithelial ovarian cancer cells. Cancer Res.

[16] Li Z, Wu G, van der Veen JN, Hermansson M, Vance DE (2014). Phosphatidylcholine metabolism and choline kinase in human osteoblasts. Biochim Biophys Acta.

[17] Trousil S, Lee P, Pinato DJ, Ellis JK, Dina R, Aboagye EO, Keun HC, Sharma R (2014). Alterations of choline phospholipid metabolism in endometrial cancer are caused by choline kinase alpha overexpression and a hyperactivated deacylation pathway. Cancer Res.

[18] Xiong J, Bian J, Wang L, Zhou JY, Wang Y, Zhao Y, Wu LL, Hu JJ, Li B, Chen SJ, Yan C, Zhao WL (2015). Dysregulated choline metabolism in T-cell lymphoma: role of choline kinase-alpha and therapeutic targeting. Blood Cancer J.

[19] Ramirez de Molina A, Gallego-Ortega D, Sarmentero J, Banez-Coronel M, Martin-Cantalejo Y, Lacal JC (2005). Choline kinase is a novel oncogene that potentiates RhoA-induced carcinogenesis. Cancer Res.

[20] Gallego-Ortega D, Gomez del Pulgar T, Valdes-Mora F, Cebrian A, Lacal JC (2011). Involvement of human choline kinase alpha and beta in carcinogenesis: a different role in lipid metabolism and biological functions. Adv Enzyme Regul.

[21] Asim M, Massie CE, Orafidiya F, Pértega-Gomes N, Warren AY, Esmaeili M, Selth LA, Zecchini HI, Luko K, Qureshi A, Baridi A, Menon S, Madhu B (2015). Choline Kinase Alpha as an Androgen Receptor Chaperone and Prostate Cancer Therapeutic Target. J Natl Cancer Inst.

[22] Granata A, Nicoletti R, Tinaglia V, De Cecco L, Pisanu ME, Ricci A, Podo F, Canevari S, Iorio E, Bagnoli M, Mezzanzanica D (2014). Choline kinase-alpha by regulating cell aggressiveness and drug sensitivity is a potential druggable target for ovarian cancer. Br J Cancer.

[23] Hernandez-Alcoceba R, Fernandez F, Lacal JC (1999). *In vivo* antitumor activity of choline kinase inhibitors: a novel target for anticancer drug discovery. Cancer Res.

[24] Lacal JC (2001). Choline kinase: a novel target for antitumor drugs. IDrugs.

[25] Yalcin A, Clem B, Makoni S, Clem A, Nelson K, Thornburg J, Siow D, Lane AN, Brock SE, Goswami U, Eaton JW, Telang S, Chesney J (2010). Selective inhibition of choline kinase simultaneously attenuates MAPK and PI3K/AKT signaling. Oncogene.

[26] Clem BF, Clem AL, Yalcin A, Goswami U, Arumugam S, Telang S, Trent JO, Chesney J (2011). A novel small molecule antagonist of choline kinase-alpha that simultaneously suppresses MAPK and PI3K/AKT signaling. Oncogene.

[27] Lacal JC, Campos JM (2015). Preclinical characterization of RSM-932A, a novel anticancer drug targeting the human choline kinase alpha, an enzyme involved in increased lipid metabolism of cancer cells. Mol Cancer Ther.

[28] Mazarico JM, Sanchez-Arevalo Lobo VJ, Favicchio R, Greenhalf W, Costello E, Carrillo-de Santa Pau E, Marques M, Lacal JC, Aboagye E, Real FX (2016). Choline Kinase Alpha (CHKalpha) as a Therapeutic Target in Pancreatic Ductal Adenocarcinoma: Expression, Predictive Value, and Sensitivity to Inhibitors. Mol Cancer Ther.

[29] Zech SG, Kohlmann A, Zhou T, Li F, Squillace RM, Parillon LE, Greenfield MT, Miller DP, Qi J, Thomas RM, Wang Y, Xu Y, Miret JJ (2016). Novel Small Molecule Inhibitors of Choline Kinase Identified by Fragment-Based Drug Discovery. J Med Chem.

[30] Schiaffino-Ortega S, Baglioni E, Mariotto E, Bortolozzi R, Serran-Aguilera L, Rios-Marco P, Carrasco-Jimenez MP, Gallo MA, Hurtado-Guerrero R, Marco C, Basso G, Viola G, Entrena A (2016). Design, synthesis, crystallization and biological evaluation of new symmetrical biscationic compounds as selective inhibitors of human Choline Kinase alpha1 (ChoKalpha1). Sci Rep.

[31] Granata A, Nicoletti R, Perego P, Iorio E, Krishnamachary B, Benigni F, Ricci A, Podo F, Bhujwalla ZM, Canevari S, Bagnoli M, Mezzanzanica D (2015). Global metabolic profile identifies choline kinase alpha as a key regulator of glutathione-dependent antioxidant cell defense in ovarian carcinoma. Oncotarget.

[32] Trousil S, Kaliszczak M, Schug Z, Nguyen Q, Tomasi G, Favicchio R, Brickute D, Fortt R, Twyman FJ, Carroll L, Kalusa A, Navaratnam N, Adejumo T (2016). The novel choline kinase inhibitor ICL-CCIC-0019 reprograms cellular metabolism and inhibits cancer cell growth. Oncotarget.

[33] Ramírez de Molina A, Sarmentero-Estrada J, Belda-Iniesta C, Tarón M, Ramírez de Molina V, Cejas P, Skrzypski M, Gallego-Ortega D, de Castro J, Casado E, García-Cabezas MA, Sánchez JJ, Nistal M (2007). Expression of choline kinase alpha to predict outcome in patients with early-stage non-small-cell lung cancer: a retrospective study. Lancet Oncol.

[34] Kwee SA, Hernandez B, Chan O, Wong L (2012). Choline kinase alpha and hexokinase-2 protein expression in hepatocellular carcinoma: association with survival. PLoS One.

[35] de la Cueva A, Ramirez de Molina A, Alvarez-Ayerza N, Ramos MA, Cebrian A, Del Pulgar TG, Lacal JC (2013). Combined 5-FU and ChoKalpha inhibitors as a new alternative therapy of colorectal cancer: evidence in human tumor-derived cell lines and mouse xenografts. PLoS One.

[36] Challapalli A, Trousil S, Hazell S, Kozlowski K, Gudi M, Aboagye EO, Mangar S (2015). Exploiting altered patterns of choline kinase-alpha expression on human prostate tissue to prognosticate prostate cancer. J Clin Pathol.

[37] Webber C, Gospodarowicz M, Sobin LH, Wittekind C, Greene FL, Mason MD, Compton C, Brierley J, Groome PA (2014). Improving the TNM classification: findings from a 10-year continuous literature review. Int J Cancer.

[38] Ramirez de Molina A, Banez-Coronel M, Gutierrez R, Rodriguez-Gonzalez A, Olmeda D, Megias D, Lacal JC (2004). Choline kinase activation is a critical requirement for the proliferation of primary human mammary epithelial cells and breast tumor progression. Cancer Res.

[39] Shah T, Wildes F, Penet MF, Winnard PT, Glunde K, Artemov D, Ackerstaff E, Gimi B, Kakkad S, Raman V, Bhujwalla ZM (2010). Choline kinase overexpression increases invasiveness and drug resistance of human breast cancer cells. NMR Biomed.

[40] Krishnamachary B, Glunde K, Wildes F, Mori N, Takagi T, Raman V, Bhujwalla ZM (2009). Noninvasive detection of lentiviral-mediated choline kinase targeting in a human breast cancer xenograft. Cancer Res.

[41] Banez-Coronel M, Ramirez de Molina A, Rodriguez-Gonzalez A, Sarmentero J, Ramos MA, Garcia-Cabezas MA, Garcia-Oroz L, Lacal JC (2008). Choline kinase alpha depletion selectively kills tumoral cells. Curr Cancer Drug Targets.

[42] Mori N, Glunde K, Takagi T, Raman V, Bhujwalla ZM (2007). Choline kinase down-regulation increases the effect of 5-fluorouracil in breast cancer cells. Cancer Res.

[43] Al-Saffar NM, Troy H, Ramirez de Molina A, Jackson LE, Madhu B, Griffiths JR, Leach MO, Workman P, Lacal JC, Judson IR, Chung YL (2006). Noninvasive magnetic resonance spectroscopic pharmacodynamic markers of the choline kinase inhibitor MN58b in human carcinoma models. Cancer Res.

[44] Kumar M, Arlauckas SP, Saksena S, Verma G, Ittyerah R, Pickup S, Popov AV, Delikatny EJ, Poptani H (2015). Magnetic resonance spectroscopy for detection of choline kinase inhibition in the treatment of brain tumors. Mol Cancer Ther.

[45] Rodriguez-Gonzalez A, Ramirez de Molina A, Fernandez F, Lacal JC (2004). Choline kinase inhibition induces the increase in ceramides resulting in a highly specific and selective cytotoxic antitumoral strategy as a potential mechanism of action. Oncogene.

[46] Sanchez-Lopez E, Zimmerman T, Gomez del Pulgar T, Moyer MP, Lacal Sanjuan JC, Cebrian A (2013). Choline kinase inhibition induces exacerbated endoplasmic reticulum stress and triggers apoptosis via CHOP in cancer cells. Cell Death Dis.

[47] Efferth T (2012). Signal transduction pathways of the epidermal growth factor receptor in colorectal cancer and their inhibition by small molecules. Curr Med Chem.

[48] Danielsen SA, Eide PW, Nesbakken A, Guren T, Leithe E, Lothe RA (2015). Portrait of the PI3K/AKT pathway in colorectal cancer. Biochim Biophys Acta.

[49] Dong P, Xu Z, Jia N, Li D, Feng Y (2009). Elevated expression of p53 gain-of-function mutation R175H in endometrial cancer cells can increase the invasive phenotypes by activation of the EGFR/PI3K/AKT pathway. Mol Cancer.

[50] Zhu G, Fan Z, Ding M, Zhang H, Mu L, Ding Y, Zhang Y, Jia B, Chen L, Chang Z, Wu W (2015). An EGFR/PI3K/AKT axis promotes accumulation of the Rac1-GEF Tiam1 that is critical in EGFR-driven tumorigenesis. Oncogene.

[51] Xu R, Shang C, Zhao J, Han Y, Liu J, Chen K, Shi W (2015). Activation of M3 muscarinic receptor by acetylcholine promotes non-small cell lung cancer cell proliferation and invasion via EGFR/PI3K/AKT pathway. Tumour Biol.

[52] Li Z, Yang Z, Passaniti A, Lapidus RG, Liu X, Cullen KJ, Dan HC (2016). A positive feedback loop involving EGFR/Akt/mTORC1 and IKK/NF-kB regulates head and neck squamous cell carcinoma proliferation. Oncotarget.

[53] Miyake T, Parsons SJ (2012). Functional interactions between Choline kinase alpha, epidermal growth factor receptor and c-Src in breast cancer cell proliferation. Oncogene.

[54] George AJ, Purdue BW, Gould CM, Thomas DW, Handoko Y, Qian H, Quaife-Ryan GA, Morgan KA, Simpson KJ, Thomas WG, Hannan RD (2013). A functional siRNA screen identifies genes modulating angiotensin II-mediated EGFR transactivation. J Cell Sci.

[55] Chua BT, Gallego-Ortega D, Ramirez de Molina A, Ullrich A, Lacal JC, Downward J (2009). Regulation of Akt(ser473) phosphorylation by choline kinase in breast carcinoma cells. Mol Cancer.

[56] Falcon SC, Hudson CS, Huang Y, Mortimore M, Golec JM, Charlton PA, Weber P, Sundaram H (2013). A non-catalytic role of choline kinase alpha is important in promoting cancer cell survival. Oncogenesis.

[57] Chen H, Hu L, Luo Z, Zhang J, Zhang C, Qiu B, Dong L, Tan Y, Ding J, Tang S, Shen F, Li Z, Wang H (2015). A20 suppresses hepatocellular carcinoma proliferation and metastasis through inhibition of Twist1 expression. Mol Cancer.

[58] Hu L, Chen L, Li L, Sun H, Yang G, Chang Y, Tu Q, Wu M, Wang H (2011). Hepatitis B virus X protein enhances cisplatin-induced hepatotoxicity via a mechanism involving degradation of Mcl-1. J Virol.

[59] Wang RY, Chen L, Chen HY, Hu L, Li L, Sun HY, Jiang F, Zhao J, Liu GM, Tang J, Chen CY, Yang YC, Chang YX (2013). MUC15 inhibits dimerization of EGFR and PI3K-AKT signaling and is associated with aggressive hepatocellular carcinomas in patients. Gastroenterology.

[60] Tang L, Tan YX, Jiang BG, Pan YF, Li SX, Yang GZ, Wang M, Wang Q, Zhang J, Zhou WP, Dong LW, Wang HY (2013). The prognostic significance and therapeutic potential of hedgehog signaling in intrahepatic cholangiocellular carcinoma. Clin Cancer Res.

[61] Hu L, Yang GZ, Zhang Y, Feng D, Zhai YX, Gong H, Qi CY, Fu H, Ye MM, Cai QP, Gao CF (2015). Overexpression of SULT2B1b is an independent prognostic indicator and promotes cell growth and invasion in colorectal carcinoma. Lab Invest.

[62] Liu N, Cui RX, He QM, Huang BJ, Sun Y, Xie D, Zeng J, Wang HY, Ma J (2013). Reduced expression of Dicer11 is associated with poor prognosis in patients with nasopharyngeal carcinoma. Med Oncol.

[63] Malinowsky K, Nitsche U, Janssen KP, Bader FG, Spath C, Drecoll E, Keller G, Hofler H, Slotta-Huspenina J, Becker KF (2014). Activation of the PI3K/AKT pathway correlates with prognosis in stage II colon cancer. Br J Cancer.

[64] Chen Z, Lu X, Wang Z, Jin G, Wang Q, Chen D, Chen T, Li J, Fan J, Cong W, Gao Q, He X (2015). Co-expression of PKM2 and TRIM35 predicts survival and recurrence in hepatocellular carcinoma. Oncotarget.

