# Supplementary material for: Overexpression of CHKA contributes to tumor progression and metastasis and predicts poor prognosis in colorectal carcinoma
Source: Oncotarget. 2016 Aug 20;7(41):66660–78. doi: 10.18632/oncotarget.11433 (PMC5341828; doi:10.18632/oncotarget.11433)
Supplement: Supplementary file 1 [file oncotarget-07-66660-s001.pdf]

Overexpression of CHKA contributes to tumor progression and metastasis and predicts poor prognosis in colorectal carcinoma

Supplementary Materials

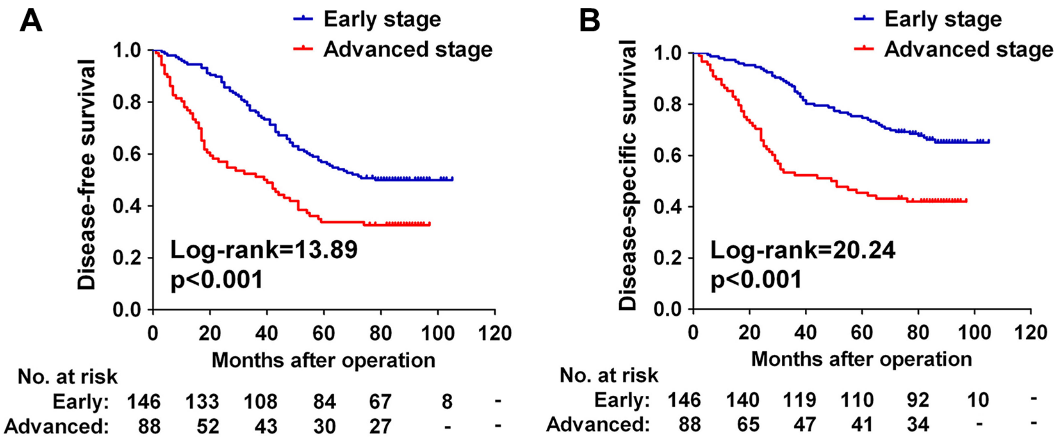

Supplementary Figure S1: Kaplan-Meier curves for disease-free survival (A) and disease-specific survival (B) of CRC patients in the study cohort according to TNM stage of the disease. The *p*-value was determined using the log-rank test. The absolute number of patients at risk in each subgroup was listed below.

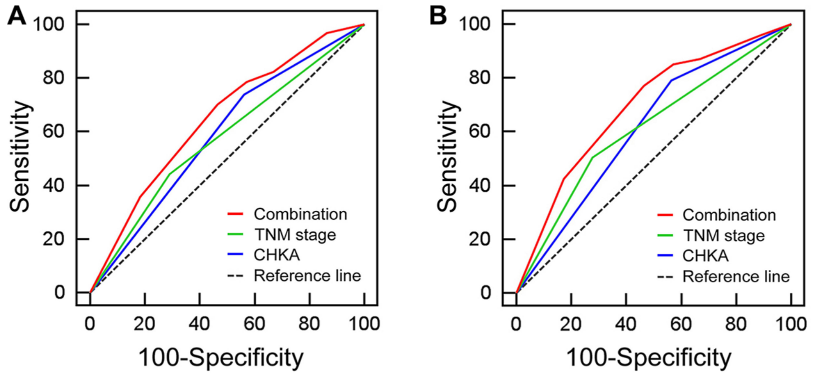

	AUC	95%CI	P value
Combination	0.658	0.583-0.709	
TNM stage	0.576	0.510-0.640	0.014
CHKA	0.589	0.525-0.653	0.007

	AUC	95%CI	P value
Combination	0.693	0.628-0.750	
TNM stage	0.613	0.547-0.675	0.006
CHKA	0.614	0.548-0.677	0.003

Supplementary Figure S2: Comparisons of the prognostic validity of the combination of CHKA expression and TNM stage model, the TNM stage alone model, and the CHKA expression alone model. Receiver operating characteristics (ROC) curves for the prediction of disease-free survival (**A**) and disease-specific survival (**B**). *P* values showed the area under the curve (AUC) of the combination model versus AUCs of the TNM stage alone model or the CHKA expression alone model.

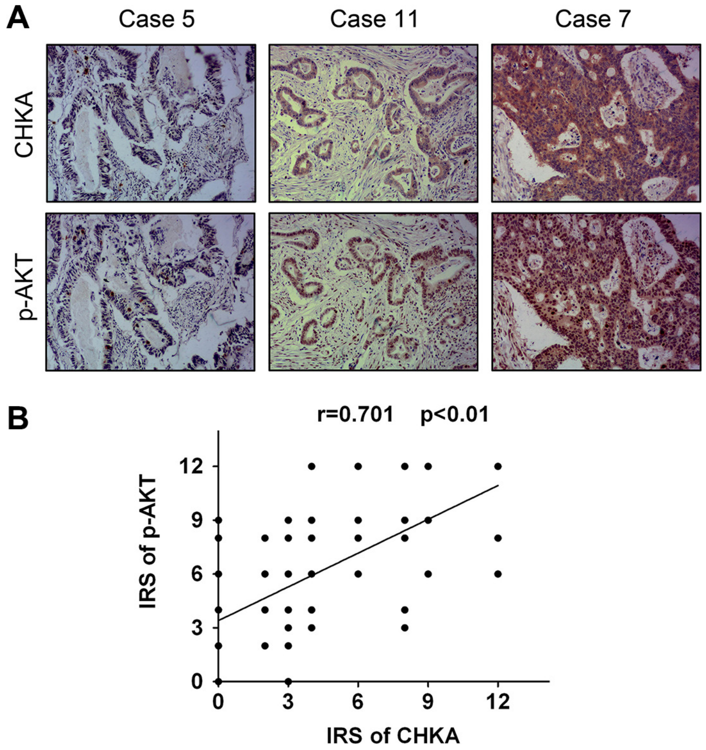

Supplementary Figure S3: (**A**) Representative immunohistochemical staining of CHKA and phospho-AKT in clinical CRC specimens. (**B**) Correlation between CHKA expression and phospho-AKT level was analyzed among 234 CRC samples using Pearson's correlation test.

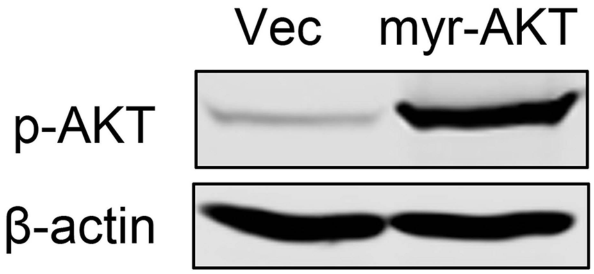

Supplementary Figure S4: HCT116 cells were transiently transfected with control vector or myr-AKT plasmid. Twenty four hours post transfection, cell lysates were harvested and subjected to Western blot assay and probed with the indicated antibodies.

Supplementary Table S1: The spearman rank correlation analysis of CHKA expression with clinicopathologic characteristics of CRC patients

Characteristics	CHKA expression	*P* value[a]
	Correlation coefficient (rs)	
**Gender**	0.059	0.371
**Age (years)**	−0.079	0.230
**Tumor location**	−0.093	0.156
**Differentiation grade**	0.104	0.113
**Tumor size (cm)**	−0.007	0.911
**Local invasion**	0.148	**0.024**
**Lymph node metastasis**	0.144	**0.028**
**TNM stage**	0.187	**0.004**
**Adjuvant chemotherapy**	0.106	0.105
**Recurrence**	0.186	**0.004**
**Death**	0.239	**< 0.001**

[a]The Spearman correlation test was used for statistical analyses. Bold type indicates statistical significance.

Supplementary Table S2: Distribution of DFS and DSS times

Variable	Mean (95% CI)	Median (95% CI)
**Complete cohort**		
DFS (months)	63.5 (58.5–68.6)	58.0 (43.7–72.3)
DSS (months)	74.3 (69.4–79.1)	NR
**Early stage group**		
DFS (months)	71.5 (65.7–77.3)	78.0 (NR)
DSS (months)	83.7 (78.5–88.9)	NR
**Advanced stage group**		
DFS (months)	47.4 (39.5–55.4)	40.0 (22.7–57.3)
DSS (months)	55.2 (47.3–63.2)	49.0 (22.3–75.7)

Abbreviations: SEM, Standard error of the mean; DSS, Disease-specific survival; DFS, Disease-free survival; NR, Not reach.

Supplementary Table S3: Distribution of DFS and DSS times stratified by tumor stage and CHKA expression

Variable	CHKA low expression subgroup		CHKA high expression subgroup	
	Mean (95% CI)	Median (95% CI)	Mean (95% CI)	Median (95% CI)
**Complete cohort**				
DFS (months)	76.7 (69.0–84.4)	NR	56.0 (49.9–62.2)	46.0 (37.4–54.6)
DSS (months)	87.5 (80.7–94.3)	NR	66.6 (60.5–72.6)	80.0 (NR)
**Early stage group**				
DFS (months)	80.5 (72.3–88.8)	NR	64.9 (57.3–72.4)	58.0 (34.4–81.6)
DSS (months)	91.5 (84.7–98.4)	NR	77.6 (70.7–84.4)	NR
**Advanced stage group**				
DFS (months)	61.3 (46.7–75.9)	54.0 (NR)	42.1 (33.2–51.1)	26.0 (11.1–40.9)
DSS (months)	70.1 (56.3–84.0)	NR	49.6 (40.6–58.7)	31.0 (14.1–47.9)

Abbreviations: SEM, Standard error of the mean; DSS, Disease-specific survival; DFS, Disease-free survival; NR, Not reach.

Supplementary Table S4: Association between intratumoral CHKA mRNA expression and clinicopathologic characteristics in the 63-patient cohort

Characteristics	No. of patients (%) (n = 63)	CHKA mRNA expression		P value[a]
		Low (%) (n = 31)	High (%) (n = 32)	
**Gender**				0.527
Female	31 (49.2)	14 (45.2)	17 (53.1)	
Male	32 (50.8)	17 (54.8)	15 (46.9)	
**Age (years)**				0.674
< 60	24 (38.1)	11 (35.5)	13 (40.6)	
≥ 60	39 (61.9)	20 (64.5)	19 (59.4)	
**Tumor location**				0.848
Rectum	44 (69.8)	22 (71.0)	22 (68.7)	
Colon	19 (30.2)	9 (29.0)	10 (31.3)	
**Differentiation grade**				0.269
Well	5 (7.9)	4 (12.9)	1 (3.1)	
Moderate	47 (74.6)	23 (74.2)	24 (75.0)	
Poor	11 (17.5)	4 (12.9)	7 (21.9)	
**Tumor size (cm)**				0.663
< 5	41 (65.1)	21 (67.7)	20 (62.5)	
≥ 5	22 (34.9)	10 (32.3)	12 (37.5)	
**Local invasion**				0.161
pT_1–T_2	20 (31.7)	13 (41.9)	7 (21.9)	
pT_3	33 (52.4)	15 (48.4)	18 (56.2)	
pT_4	10 (15.9)	3 (9.7)	7 (21.9)	
**Lymph node metastasis**				**0.015**
Negative	35 (55.6)	22 (71.0)	13 (40.6)	
Positive	28 (44.4)	9 (29.0)	19 (59.4)	
**TNM stage**				**< 0.001**
I	15 (23.8)	14 (45.2)	1 (3.1)	
II	20 (31.7)	8 (25.8)	12 (37.5)	
III	28 (44.5)	9 (29.0)	19 (59.4)	
**Adjuvant chemotherapy**				**0.002**
No	32 (50.8)	22 (71.0)	10 (31.3)	
Yes	31 (49.2)	9 (29.0)	22 (68.7)	
**Recurrence**				0.525
No	53 (84.1)	27 (87.1)	26 (81.3)	
Yes	10 (15.9)	4 (12.9)	6 (18.7)	
**Death**				0.668
No	58 (92.1)	29 (93.5)	29 (90.6)	
Yes	5 (7.9)	2 (6.5)	3 (9.4)	

[a] Pearson chi-square test was used for comparison between subgroups. Bold type indicates statistical significance.
